# The microbiome–nitric oxide axis in cancer immunotherapy: a targeted qualitative systematic review of mechanistic and translational evidence

**DOI:** 10.3389/fimmu.2026.1904698

**Published:** 2026-09-24

**Authors:** Michael Enwere, Nathan S. Bryan, Adamu Onu, Mercy Neufeld, Mohamed Toufic El-Hussein, Ibrahim Yakub, Charles Omale, Ogonna Onyedike, Terkaa Atim, Olukayode Agbeyomi, Emmanuel Davies, Ujuaku E. Okeke, Laurens Holmes

**Affiliations:** 1 Global Health Equity Foundation (GHEF), Bear, DE, United States; 2 PrimeLife360 Wellness, London, United Kingdom; 3 Bryan Therapeutics Inc., Austin, TX, United States; 4 Institute of Medical Sciences Africa, Abuja, Nigeria; 5 Swedish Medical Center Colorado, Englewood, CO, United States; 6 AgeCare Research Chair in Older Adults Health, School of Nursing and Midwifery, Faculty of Health, Community and Education, Mount Royal University, Calgary, AB, Canada; 7 Well-Life Hospital, Abuja, Nigeria; 8 Royal Eye Hospital & Medical Centre, Abuja, Nigeria; 9 Friendly Hospital, Enugu, Nigeria; 10 Department of Surgery, Faculty of Clinical Sciences, College of Health Sciences, University of Abuja, Abuja, Nigeria; 11 Department of Public Health, Babcock University, Ilishan-Remo, Ogun State, Nigeria; 12 Department of Preventive and Social Medicine, University of Port Harcourt School of Public Health, Choba, Nigeria; 13 Public Health Informatics & Data Exchange, Texas Department of State Health Services, Austin, TX, United States; 14 Public Health Institute, Florida Agricultural and Mechanical University (FAMU), Tallahassee, FL, United States

**Keywords:** *Akkermansia muciniphila*, cancer immunotherapy, gut microbiome, immune checkpoint inhibitors (ICI), microbiome–immune crosstalk, nitric oxide (NO), short-chain fatty acids (SCFAs), tumor microenvironment (TME)

## Abstract

**Background:**

Immune checkpoint inhibitors (ICIs) have transformed cancer treatment, but responses vary substantially among patients. Clinical studies have associated features of the gut microbiome with ICI outcomes, while experimental evidence suggests that microbial metabolites and nitric oxide (NO)-related pathways may influence antitumor immunity and tumor vascular biology. This qualitative systematic review evaluated the evidence supporting a proposed microbiome–NO–ICI framework.

**Methods:**

Five databases were searched for peer-reviewed studies published between January 2000 and August 2025. Fifty-four distinct primary studies met the eligibility criteria, comprising 21 clinical studies, 19 animal studies and 14 *in vitro* mechanistic studies. Evidence was synthesized narratively according to study type and thematic domain.

**Results:**

Several clinical cohorts associated enrichment of *Akkermansia muciniphila*, *Faecalibacterium prausnitzii*, *Bifidobacterium* species and other short-chain fatty acid–producing taxa with favorable ICI outcomes; however, the reported microbial signatures varied across cohorts and analytical platforms. Microbial-transfer experiments and early non-randomized fecal microbiota transplantation studies provide proof-of-concept evidence that microbiome composition can influence antitumor immune responses, although the clinical intervention evidence remains preliminary. Animal and cellular studies indicate that microbial metabolites, host nitric oxide synthase pathways and reactive nitrogen signaling can affect immune-cell function, vascular regulation and tumor hypoxia. Direct clinical evidence linking microbiota-mediated nitrate reduction or dietary nitrate supplementation to improved ICI outcomes is lacking. Human dietary nitrate studies conducted outside oncology demonstrate increased circulating nitrate and nitrite and measurable vascular effects but do not establish anticancer or ICI efficacy.

**Conclusion:**

The microbiome–NO–ICI axis should be regarded as a testable integrative model linking microbial ecology, immune regulation and tumor vascular biology. Current evidence supports biological plausibility but is insufficient to establish NO- or microbiome-directed interventions as effective adjuncts to ICI therapy. Prospective mechanistic studies and biomarker-driven clinical trials are required before therapeutic translation.

## Introduction

The gut microbiome is increasingly recognized as an important regulator of systemic immunity, inflammation, and treatment response in cancer. At the same time, immune checkpoint inhibitors (ICIs) targeting programmed cell death protein 1 (PD-1), programmed death-ligand 1 (PD-L1), and cytotoxic T-lymphocyte-associated protein 4 (CTLA-4) have reshaped cancer therapy by restoring antitumor immune recognition and promoting tumor-cell killing (1, 2). CTLA-4 is expressed on activated conventional CD4^+^ and CD8^+^ T-cell subsets and constitutively at high levels on regulatory T cells; its expression is therefore not confined to CD8^+^ T cells. Agents such as ipilimumab, pembrolizumab, and nivolumab have shown efficacy in malignancies including melanoma, non-small-cell lung cancer, and lymphomas, with associated improvements in survival and quality of life (1, 3). Despite these therapeutic advances, the cancers most commonly treated with ICIs such as non-small cell lung cancer (NSCLC), melanoma, and renal cell carcinoma remain major global health burdens (4). Lung cancer alone accounts for approximately 1.8 million deaths annually, with NSCLC comprising nearly 85% of cases (4). Melanoma incidence has risen steadily, reaching more than 325,000 new cases and 57,000 deaths worldwide in 2020 (5), while renal cell carcinoma accounts for roughly 430,000 new diagnoses each year (6). While real-world studies confirm that Immune Checkpoint Inhibitors (ICIs) have improved survival, overall response rates typically remain modest at approximately 29% (7, 8).

This underscores the critical need to identify modifiable biological determinants of treatment response. Emerging evidence suggests that microbiome-associated metabolites and NO-related pathways may modulate T-cell function and tumor-microenvironmental biology (9–11).

Despite these advances, responses to ICIs remain heterogeneous. Patient outcomes are shaped by tumor-intrinsic features like tumor mutational burden and PD-L1 expression, as well as host-related factors including somatic and germline genomic profiles, immune system status, and comorbid conditions (12–14). Age-related inflammatory andmetabolic disorders further compound treatment variability, and the immune microenvironment, shaped by prior infections, therapies, and immune history plays a critical role in dictating therapeutic success (15).

Among emerging host-related determinants, the gut microbiome has gained recognition as a potential modulator of cancer-immunotherapy outcomes. Numerous studies have shown that microbial composition, diversity, and functional metabolites influence ICI efficacy and toxicity profiles (16, 17). Clinical and preclinical evidence indicates that differences in gut microbial composition may contribute to variability in response and toxicity associated with immune checkpoint inhibitors, although the causal mechanisms and predictive value of individual microbial signatures remain incompletely established (18, 19). Enrichment of specific taxa, such as Faecalibacterium prausnitzii, Bifidobacterium breve, B. longum, and Akkermansia muciniphila has been linked to improved ICI responsiveness and prolonged survival (20–23). For instance, A. muciniphila abundance is associated with favorable immune infiltration and therapeutic outcomes in lung cancer patients (24), and oral supplementation with live A. muciniphila restores anti-PD-1 efficacy in antibiotic-treated or germ-free mice by enhancing CD8^+^ T cell activation and IL-12–mediated Th1 polarization (25, 26). Understanding the microbiome–nitric oxide axis holds significant translational potential beyond its mechanistic importance. Knowledge gained from this interaction could guide the development of microbiota-targeted interventions, including fecal microbiota transplantation, live biotherapeutics containing beneficial bacterial strains, and dietary modifications that promote microbial nitrate reduction and endogenous NO synthesis. These approaches provide testable strategies for future mechanistic and biomarker-guided studies, but combined microbiome- and NO-directed interventions have not been shown to improve ICI efficacy and should remain investigational.

The mechanistic underpinnings of these effects involve multiple immunomodulatory pathways. Gut bacteria support ICI efficacy by enhancing mucosal barrier integrity, promoting dendritic cell activation, increasing T-cell priming, and producingimmunoregulatory short-chain fatty acids (SCFAs) such as acetate and butyrate (17, 27, 28). Butyrate, in particular, has been shown to improve chemotherapy and immunotherapy outcomes by modulating histone acetylation at the Pdcd1 and Cd28 promoters and enhancing T-cell receptor signaling in cytotoxic CD8^+^ T cells (29). These microbial-derived metabolites influence immune homeostasis, cytokine signaling, and epigenetic regulation, contributing to both antitumor immunity and the mitigation of immune-related adverse events (21, 22).

These findings support the emerging concept of “oncomicrobiotics” microbiota-targeting strategies designed to enhance cancer immunotherapy by manipulating specific microbial communities or their metabolites (27). However, the immunological effects of these interventions remain context-dependent, influenced by microbial concentration, cancer type, and host immune status. A deeper understanding of these complex interactions is essential to refine patient stratification, identify predictive biomarkers, and design personalized microbiota-informed combination therapies (22, 23).

Besides these immunomodulatory effects, certain gut microbes also influence antitumor immunity through vascular and metabolic pathways, particularly by modulating nitric oxide (NO) signaling (30–32). For instance, Akkermansia muciniphila restores responsiveness to anti-PD-1 therapy in germ-free or antibiotic-treated hosts by promoting Th1 polarization and CD8^+^ T cell infiltration (33–35), while Ruminococcus species support mucosal integrity and immune conditioning through mucin degradation and short-chain fatty acid (SCFA) production (36).

Nitric oxide (NO) is a pleiotropic signaling molecule whose effects on tumor biology vary with its cellular source, local flux, duration of exposure, spatial distribution, and the surrounding redox environment. It is produced enzymatically by nitric oxide synthases and through the oxygen-independent nitrate–nitrite–NO pathway, which is supported by nitrate-reducing microorganisms in the oral cavity and gastrointestinal tract (37–40). Taxa such as Veillonella, Actinomyces, Bacteroides, and Neisseria can reduce dietary nitrate to nitrite, thereby contributing to systemic NO generation through the enterosalivary pathway (41–43). Under low-oxygen and acidic conditions, nitrite can be reduced to NO through oxygen-independent pathways. However, whether oral or gastrointestinal microbial nitrate reduction meaningfully alters intratumoral NO bioavailability or responses to immune checkpoint inhibition has not been established.

NO cannot be assigned a uniformly pro-tumor or antitumor role on the basis of concentration alone. Its biological effects are determined jointly by cellular source, local flux, duration and spatial distribution of exposure, oxygen tension, tumor type, and the surrounding redox environment (42, 44). Depending on these conditions, NO may preserve or normalize vascular function, alter tumor perfusion, facilitate antitumor immune-cell trafficking, and induce tumor-cell cytotoxicity. In other settings, it may promote pathological angiogenesis, immune suppression, DNA damage and genomic instability, or tumor progression (9, 10, 42, 44, 45).

Taken together, these observations argue against treating either augmentation or inhibition of NO-related pathways as uniformly beneficial. Any therapeutic approach would need to account for the tumor type, cellular source of NO, local biological context, and intended treatment effect. This context dependence is particularly relevant to NOS-directed strategies, because NO signaling intersects with epithelial–mesenchymal transition, post-translational regulation, vascular function, and T-cell trafficking. NOS inhibition may therefore offer therapeutic benefit in selected settings, but it should be regarded as a context-dependent investigational approach rather than a universally applicable means of improving treatment efficacy (46).

At the microbial level, disruption of nitrate-reducing communities through antibiotics, low-nitrate dietary exposure, or chronic illness may reduce systemic NO bioavailability (47, 48). Whether such changes also diminish responsiveness to cancer therapy, however, remains uncertain and requires direct investigation. This qualitative systematic review integrates microbiota-mediated NO signaling with the broader evidence concerning ICI responsiveness, two areas that have generally been examined separately. Previous reviews have generally considered the role of the microbiome in immunotherapy and the effects of NO in tumor biology as separate areas of investigation. The present synthesis brings these literatures together through a proposed microbiome–NO–ICI framework encompassing tumor vascular regulation, immune-cell infiltration, and hypoxic signaling. The evidence remains limited by inconsistent microbiome-profiling approaches, variation in NO measurement, and the scarcity of longitudinal mechanistic studies in humans.

Additionally, most available data originate from Western populations, potentially limiting broader applicability across diverse global populations.

These methodological and knowledge gaps underscore the need for integrated clinical, preclinical and mechanistic studies to clarify the proposed microbiome–NO–ICI relationship.

Current evidence provides a rationale for investigating whether nitrate-reducing microbial functions influence immune and vascular phenotypes relevant to ICI response. However, direct evidence that modifying these functions improves clinical ICI outcomes remains limited.

Identifying key microbial taxa involved in this pathway and elucidating their mechanistic roles in tumor immunity are critical next steps. This review was designed to examine a prespecified mechanistic framework, rather than to catalog or rank all microbiome-based predictors of ICI outcomes. The prominence of individual taxa in the synthesis should therefore be understood in the context of the review’s focused microbiome–NO–ICI framework.

To visually synthesize the conceptual framework emerging from this qualitative systematic review, **Figure 1** integrates two related but distinct evidence streams. The first comprises microbiome–immune mechanisms involving microbial metabolites, mucosal-barrier function and immune priming, including pathways associated with taxa such as Akkermansia muciniphila, Faecalibacterium spp. and Ruminococcus spp. The second represents proposed cross-domain interactions between microbial functions and microbiota-supported or host nitric oxide (NO) pathways that may influence vascular and immune phenotypes within the tumor microenvironment. These pathways include short-chain fatty acid production, mucin utilization and barrier support, immune-modulatory metabolite signaling, and microbial nitrate–nitrite metabolism; however, these functions should not be attributed uniformly to all taxa represented in the figure. Importantly, the connections integrating microbiome-related processes with NO signaling and ICI-related outcomes are supported predominantly by indirect mechanistic or preclinical evidence and should therefore be interpreted as a hypothesis-generating framework rather than a demonstrated causal microbiome–NO–ICI pathway. These microbial metabolites act as signaling intermediates that interact with host nitric oxide synthase pathways, including endothelial nitric oxide synthase (eNOS) and inducible nitric oxide synthase (iNOS), thereby modulating redox balance, tumor vascular tone, immune-cell trafficking, and hypoxic stress within the tumor microenvironment. Through these interconnected metabolic and immunovascular processes, the microbiome–NO axis may influence antigen presentation, CD8^+^ T-cell activity, and the responsiveness or resistance of tumors to immune checkpoint inhibitor therapy.

**Figure 1:**
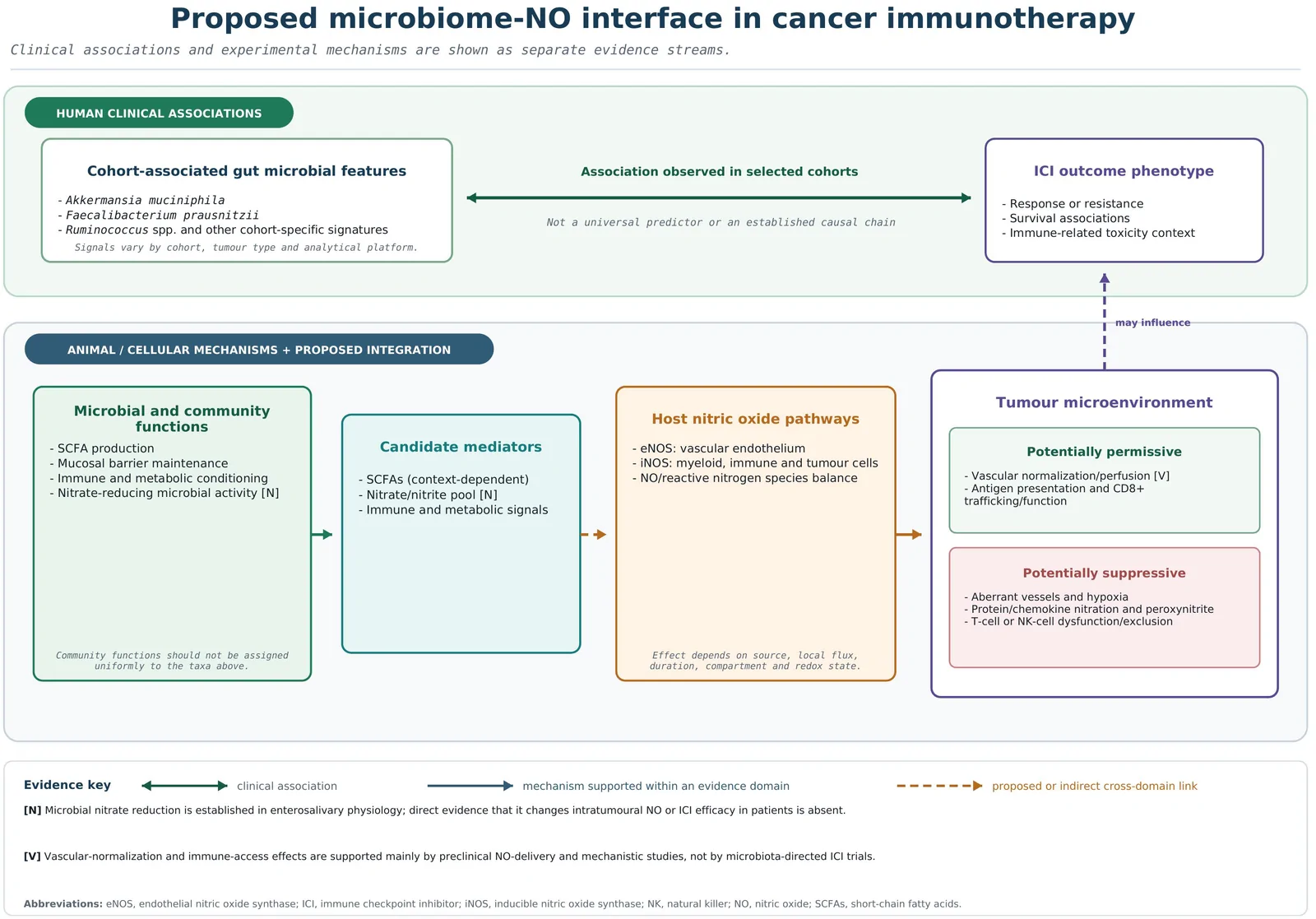
Proposed microbiome–nitric oxide interface in cancer immunotherapy. Human clinical studies have associated selected gut microbial features—including Akkermansia muciniphila, Faecalibacterium prausnitzii and Ruminococcus spp.—with immune checkpoint inhibitor outcomes; however, these signatures vary across cohorts, tumor types and analytical platforms and are not established as universal predictors or causal determinants. Animal and cellular studies indicate that microbial functions, including short-chain fatty acid production, mucosal-barrier maintenance and nitrate-reducing activity, can influence immune and metabolic signaling. These functions may intersect with host eNOS- and iNOS-dependent pathways, although a microbiome–nitrate–NO–ICI pathway has not been demonstrated directly in patients. NO effects are context-dependent and may support vascular normalization, immune-cell trafficking and effector activity or, conversely, promote hypoxia, nitration and immune-cell dysfunction or exclusion. Solid arrows represent mechanisms supported within an evidence domain, dashed arrows represent proposed or indirect cross-domain links, and the bidirectional arrow represents clinical association without established causality.

Building upon this mechanistic framework, **Figure 2** highlights potential translational strategies that could be used to therapeutically target the microbiome–NO axis in cancer immunotherapy. These strategies include microbiome and NO-pathway biomarker profiling for patient stratification, dietary and metabolic interventions aimed at modulating microbial function, microbiota replacement approaches such as fecal microbiota transplantation or defined microbial consortia, and rational integration of microbiome-directed interventions with immune checkpoint blockade. Together, these translational pathways illustrate how modulation of microbial ecology and NO signaling could support precision immuno-oncology strategies designed to enhance treatment responsiveness and overcome resistance to immune checkpoint inhibitors.

**Figure 2:**
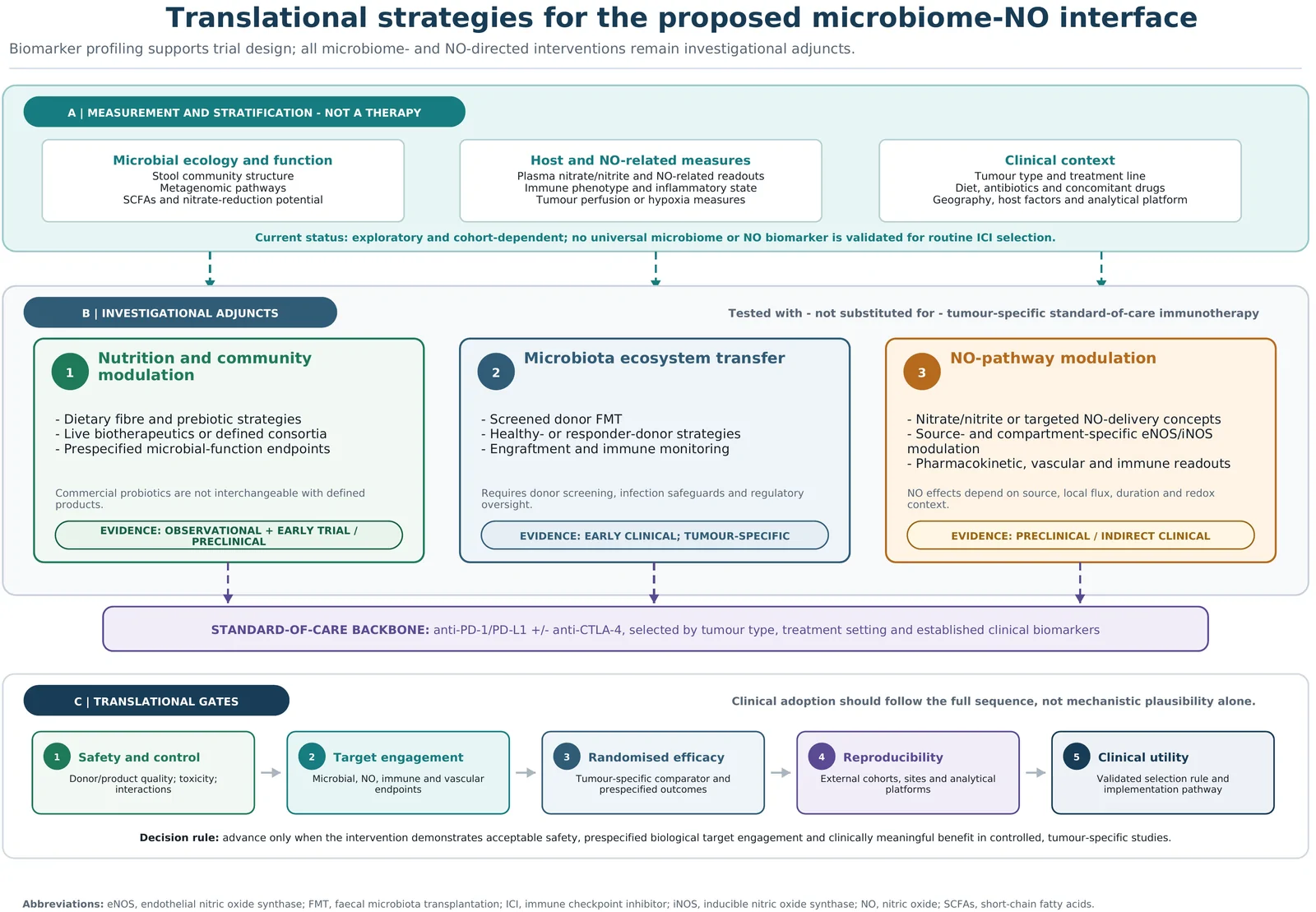
Translational strategies for the proposed microbiome–nitric oxide interface in cancer immunotherapy.

The constituent pathways of this microbiome–NO–ICI axis, along with their supporting evidence and translational implications, are synthesized in **Table 1**, which integrates mechanistic insights from preclinical models with observations from clinical studies.

**Table 1:** Mechanistic pathways and evidence supporting the microbiome–nitric oxide–immune checkpoint inhibitor axis.

Pathway/target	Key microbial taxa & metabolites	Proposed mechanism of action	Supporting preclinical evidence	Associated clinical outcomes in ICI therapy	Potential intervention strategy
*Immune Priming & T-cell Activation*	*A. muciniphila*, *Bifidobacterium* spp.	• Dendritic cell maturation & IL-12 secretion • Th1 polarization • ↓PD-1/PD-L1 expression on T cells/tumors	Oral *A. muciniphila* restores anti-PD-1 efficacy in GF mice (24, 29); FMT from responders transfers sensitivity (45).	Enriched in NSCLC/melanoma responders; correlates with ↑CD8+ TILs and ↑IFN-γ signature (24, 47, 102).	Defined live biotherapeutics (e.g., *A. muciniphila* capsules); FMT from validated responders.
*SCFA-Mediated Epigenetic & Vascular Modulation*	*Ruminococcus* spp., *F. prausnitzii* (Butyrate)	• HDAC inhibition → acetylation at *Pdcd1*/*Cd28* loci • eNOS activation → ↑NO → vascular normalization	Butyrate + anti-PD-1 enhances tumor regression & CD8+/Treg ratio in CT26 models (71, 75); improves T-cell trafficking (72).	High fecal butyrate/butyrateproducers correlate with prolonged PFS (4-9 months) in melanoma/NSCLC (48, 77).	High-fiber/prebiotic diets; postbiotic butyrate formulations; pharmacologic HDAC inhibitors.
*Nitrate-Nitrite-NO Pathway (Microbial)*	*Veillonella*, *Neisseria*, *Actinomyces* spp.	• Nitrate reductase activity → systemic nitrite/NO pool Compensatory NO source under tumor hypoxia	Dietary nitrate improves tumor perfusion & reduces hypoxia in preclinical models; synergizes with anti-PD-1 (99, 100, 140).	(Indirect) Antibiotic use depletes nitratereducers and worsens ICI response (24); direct clinical correlations pending.	Dietary nitrate (beetroot juice); prebiotics for nitratereducer enrichment.
*Integrated Axis* *Effect: Vascular* *Normalization & Immune Access*	*A. muciniphila* + *Ruminococcus* spp. (SCFAs) + Nitrate Reducers	• Combined immune activation + SCFA/NO-driven vascular remodeling → ↑perfusion, ↓hypoxia, ↑immune infiltration	Co-administration shows additive effects on tumor control and CD8+ T-cell infiltration vs. single interventions (29, 99).	Patients with combined "favorable" signatures show superior ORR and OS (47, 103).	Multi-strain consortia; combined dietary nitrate + prebiotic/probiotic regimens.

HDAC, histone deacetylase; eNOS, endothelial nitric oxide synthase; FMT, fecal microbiota transplantation; GF, germ-free; TILs, tumor-infiltrating lymphocytes; PFS, progressionfree survival; ORR, objective response rate; OS, overall survival; NSCLC, non-small cell lung cancer.↑, increased/upregulated; ↓, decreased/downregulated; →, leads to/promotes or denotes the indicated mechanistic relationship.

Biomarker profiling is presented as a measurement and patient-stratification framework rather than as a therapeutic intervention. Candidate adjunctive strategies include dietary fiber and prebiotic approaches, defined live biotherapeutics, screened-donor fecal microbiota transplantation (FMT), rational microbial consortia and context-specific modulation of nitric oxide (NO) pathways. The supporting evidence remains heterogeneous: dietary findings are predominantly observational; live biotherapeutic and FMT studies provide early, tumor-specific clinical signals; and NO-directed approaches remain supported mainly by pharmacokinetic, vascular and preclinical oncology evidence. None of these interventions should be considered a substitute for tumor-specific standard-of-care immune checkpoint blockade. Clinical translation requires sequential demonstration of product or donor safety, biological target engagement, randomized efficacy, external reproducibility and clinical utility. Microbiome and NO biomarkers remain exploratory and are not validated for routine selection of patients for ICI therapy.

## Methods

### Study design

The Qualitative Systematic Review (QualSR) evaluated a defined mechanistic framework linking gut microbial taxa and functions—including evidence concerning Akkermansia muciniphila and Ruminococcus spp.—with immune checkpoint inhibitor response, nitric oxide signaling and related immune–vascular pathways. The review was not designed as an exhaustive assessment or comparative ranking of all microbiome correlates of ICI outcomes. This qualitative systematic review was conducted using a structured review methodology and is reported in accordance with the Preferred Reporting Items for Systematic Reviews and Meta-Analyses 2020 statement. The completed PRISMA 2020 checklist is provided in Supplementary File S3.

The QualSR specifically aimed to answer five central research questions:

1. Within the defined microbiome–NO–ICI framework, what clinical, animal and *in vitro* evidence links selected microbial taxa and microbial functions to ICI response, and how consistent are the observed associations or mechanisms across evidence strata?1.
2. How do *A. muciniphila* and *Ruminococcus* species mechanistically influence ICI efficacy?
3. What role does nitric oxide signaling—both enzymatic and microbiota-derived—play in modulating antitumor immune responses?
4. Are there identifiable pathways through which gut microbiota affect vascular normalization, immune cell infiltration, or tumor hypoxia in ICI-treated patients?
5. Can modulation of the nitrate–nitrite–NO pathway enhance the clinical benefits of immunotherapy?

### Protocol and registration

This review was not prospectively registered, and no formal protocol was published before study selection and data extraction began. This is acknowledged as a methodological limitation. To maximize transparency regarding the surviving review records, the manuscript reports the databases searched, recoverable search concepts, eligibility framework, study-selection procedures, evidence-stratum classification, methodological-appraisal methods, reconstructed full-text exclusion reasons and PRISMA 2020 flow diagram. The exact database-specific search strings as originally executed and the exact final search dates were not fully recoverable from the retained records and are therefore not presented as a verbatim reconstruction of the historical electronic searches.

### Search strategy

A structured literature search was conducted in five major databases: PubMed, EMBASE, Scopus, Web of Science, and Cochrane Library, covering publications from January 2000 to August 2025. The surviving documentation established the databases, search period and principal search concepts but did not permit reliable reconstruction of every database-specific string or exact execution date. The search terms reported below therefore describe the recoverable search framework rather than a verbatim historical search log.

Search terms included combinations of:

- “gut microbiota” OR “intestinal flora” OR “microbiome”
- “immune checkpoint inhibitors” OR “PD-1” OR “PD-L1” OR “CTLA-4”
- “*Akkermansia muciniphila*” OR “*Ruminococcus*” OR “commensal bacteria”
- “nitric oxide” OR “nitrate reduction” OR “tumor perfusion” OR “vascular modulation”

Boolean operators (AND/OR) and MeSH terms were used to refine results. Only peer-reviewed, English-language publications were considered.

### Eligibility criteria and evidence-stratum classification

Eligible records comprised peer-reviewed primary research studies published in English between 1 January 2000 and August 2025. The review was intended to bring together clinical observations and preclinical and mechanistic evidence relevant to the proposed microbiome–nitric oxide–immune checkpoint inhibitor (microbiome–NO–ICI) framework. Studies were therefore classified into three distinct evidence strata: human clinical studies, animal studies, and *in vitro* mechanistic studies. The eligibility framework was clarified during revision to resolve inconsistencies in the original manuscript regarding the treatment of animal and *in vitro* mechanistic evidence. Because the review was not prospectively registered and complete contemporaneous screening documentation was unavailable, the eligibility criteria presented here should not be interpreted as having been prospectively specified in their present form. The revision formalized the evidence-stratum framework used for the current synthesis, and this retrospective clarification is acknowledged as a methodological limitation.

Human studies were eligible when they evaluated associations between the gut microbiome, microbial taxa, microbial functional pathways, or microbiome-derived metabolites and outcomes related to immune checkpoint blockade targeting PD-1, PD-L1, or CTLA-4.

In human studies, “ICI outcome” was used as an umbrella term rather than as a single interchangeable endpoint. Objective response referred to complete or partial radiographic response according to RECIST, iRECIST or the criteria specified by the original investigators. Clinical benefit was retained only when defined by the primary study and was not treated as synonymous with objective response. Stable disease, progression-free survival, overall survival, toxicity and immune phenotypes were extracted separately. Because outcome definitions, tumor types and treatment regimens differed substantially, response categories were not quantitatively pooled across studies.

Animal studies were eligible when they experimentally investigated a mechanistic component relevant to the review framework, including microbiome- or NO-related determinants of antitumor immunity, tumor-microenvironmental or vascular biology, immune-cell infiltration, or responses to PD-1, PD-L1 or CTLA-4 blockade. Direct administration of an ICI was not required where the study addressed a defined mechanistic component of the proposed framework; such studies were classified as mechanistic supporting evidence and were not interpreted as direct evidence that the mechanism improves ICI efficacy.

*In vitro* mechanistic studies constituted a separate evidence stratum and were eligible only when they directly interrogated a mechanistic link relevant to the microbiome–NO–ICI framework. These studies included investigations of microbial taxa or microbial metabolites; nitrate–nitrite–NO or nitric oxide synthase signaling; short-chain fatty acids or other microbiota-derived metabolites; immune-cell activation or suppression; endothelial or vascular responses; and tumor-cell phenotypes directly relevant to antitumor immunity or ICI response. Their role in the review was mechanistic: they were included to clarify biological pathways and were not interpreted as direct evidence of clinical ICI efficacy. Direct exposure to an ICI was therefore not mandatory for *in vitro* eligibility when the experiment directly interrogated a mechanistic pathway incorporated into the review framework.

Reviews, systematic reviews, meta-analyses, editorials, commentaries, letters and other non-primary publications were excluded from the primary evidence synthesis. Conference abstracts without a corresponding full peer-reviewed report were similarly ineligible for inclusion in the formal evidence base. Where an abstract was cited solely to acknowledge preliminary or emerging findings, it was explicitly identified as a conference report and was not included in study counts, data extraction, risk-of-bias assessment or the conclusions of the review. Non-English-language publications were also excluded, as were studies that lacked a relevant microbiome, microbial-metabolite, nitric oxide, immune, vascular, tumor-microenvironment or ICI-related endpoint. In vitro studies without a direct mechanistic connection to the defined review questions were not eligible.

Evidence from human, animal, and in vitro studies was extracted and synthesized within its respective stratum before comparison across the review’s thematic domains. This distinction was maintained throughout the analysis. Findings from in vitro studies were used to inform biological plausibility and pathway interpretation, but they were not treated as equivalent to evidence of therapeutic efficacy in animal models or clinical benefit in patients receiving immune checkpoint inhibitors.

### Study selection and data extraction

The original full-text selection ledger was not available when the initial manuscript and supplementary documentation were prepared, and the full-text exclusion reasons were therefore initially reported as unavailable. During revision, the ledger was reconstructed from the retained review records, manuscript citation corpus, reference list, included-study inventory and accessible full-text reports. The reconstructed ledger reproduced the original full-text eligibility decisions and exclusion categories. To verify its accuracy, a second reviewer independently reviewed all 164 full-text reports against the eligibility criteria and checked the reconstructed inclusion/exclusion decisions and the primary reason assigned to each excluded report. Disagreements identified during this verification process were resolved through discussion and consensus, with third-reviewer adjudication where required. The verified ledger was then used to regenerate the PRISMA 2020 flow diagram.

Data were extracted using a standardized form, capturing:

- Study design and model (clinical or preclinical)
- Microbial species studied
- ICI regimen used
- Microbiome analysis methods (e.g., 16S rRNA sequencing, shotgun metagenomics)
- Measures of immune response (e.g., CD8+ T cell activation, cytokine production)
- Vascular/perfusion markers (e.g., NO bioavailability, hypoxia levels)
- Intervention strategies (e.g., FMT, probiotics, dietary nitrate)

During revision, the included-study inventory and bibliographic records were reconciled against the corresponding primary reports and **Supplementary Materials**. Study characteristics were re-extracted, and information that remained unavailable after review of the full report and any accompanying **Supplementary Material** was recorded as not reported (NR), without estimation or imputation. The reconciled citations and extracted data were independently verified by a second reviewer, with disagreements resolved by consensus.

Following this reconciliation, each included study was assigned to one of three evidence strata—human clinical, animal preclinical or in vitro mechanistic—according to its primary experimental design. For publications containing more than one type of evidence, the principal dataset contributing to the synthesis determined the study-level classification, while relevant findings from secondary experimental components were extracted and identified separately. The three evidence streams were kept distinct throughout data extraction, synthesis and interpretation to avoid conflating clinical associations with in vivo experimental findings or in vitro evidence of mechanistic plausibility. Supplementary **Supplementary Table S1** presents the characteristics of the included studies, including study design, clinical population or experimental model, microbiome or nitric oxide exposure, immune checkpoint inhibitor regimen where applicable, analytical methods, principal outcomes and assigned evidence domain.

### Quality and bias assessment

During revision, two reviewers independently appraised every included study using the design-appropriate instruments specified below. Each reviewer recorded domain-level judgments separately before the results were compared. Disagreements were resolved through discussion and consensus, with third-reviewer adjudication where necessary. The completed reviewer-specific forms and consensus decisions were retained in the review archive.

Randomized clinical trials were assessed using the revised Cochrane Risk of Bias tool (RoB 2), and non-randomized intervention studies with ROBINS-I. Observational cohort studies were evaluated using the Newcastle–Ottawa Scale; these assessments were reported as measures of methodological quality rather than as formal domain-based risk-of-bias judgments. Animal experiments were assessed with the SYRCLE Risk of Bias tool.

No single validated instrument is universally accepted for the heterogeneous body of in vitro mechanistic research included in this review. We therefore used a structured framework to evaluate the methodological quality of the in vitro mechanistic studies. The assessment covered the adequacy of biological and vehicle controls, independent replication, characterization of the microbial product or NO exposure, blinded or automated outcome assessment where applicable, completeness of statistical reporting, and possible selective outcome reporting. STROBE and relevant omics-reporting guidance were used to examine reporting completeness, not to determine risk of bias.

### Data synthesis approach

Throughout this review, evidence from human clinical studies, animal experiments and in vitro mechanistic studies was interpreted within its appropriate evidentiary context. Each evidence stream was synthesized separately before findings were integrated across domains. Observational associations in human studies were not taken to establish causality, nor were findings from animal or in vitro models treated as direct evidence of clinical efficacy. Instead, convergence across evidence strata was used to assess biological plausibility. The proposed microbiome–NO–ICI framework should therefore be viewed as an integrative, hypothesis-generating mechanistic model, rather than as a clinically validated causal pathway or therapeutic strategy.

Given substantial heterogeneity in study design, cancer type, microbiome profiling methodology, nitric oxide assessment, experimental model, and outcome definition, a quantitative meta-analysis was not considered appropriate. Accordingly, a narrative thematic synthesis was undertaken. Findings were grouped into five thematic domains aligned with the defined review questions:

1. Microbial Taxa and ICI Response

Identification of commensals—particularly *A. muciniphila* and *Ruminococcus* spp.—associated with improved ICI efficacy.

2. Mechanistic Insights

Microbial pathways influencing mucosal integrity, SCFA signaling, antigen presentation, and T cell priming.

3. NO Signaling Pathways

Role of nitrate–nitrite–NO axis and microbial modulation of vascular or immune parameters affecting the TME.

4. Tumor Microenvironment Remodeling

Evidence on microbial or NO-driven changes in tumor perfusion, immune cell infiltration, or oxygenation.

5. Intervention Strategies

Studies involving dietary, probiotic, or FMT-based modulation of microbial communities and NO dynamics.

Quantitative results (e.g., NO levels, tumor regression rates) were summarized descriptively. This variability precluded statistical pooling and necessitated a qualitative, narrative synthesis to ensure accurate interpretation of findings.

## Results and discussion

### Study selection and characteristics

The retained review records documented 1,236 identified records, comprising 1,206 database records and 30 records identified through other methods. After removal of 312 duplicates, 924 records underwent title and abstract screening, of which 760 were excluded. All 164 reports sought for retrieval were obtained and assessed for eligibility in full text.

Following independent revision-stage verification of the reconstructed full-text ledger, 110 reports were excluded for the mutually exclusive reasons shown in **Figure 3**, leaving 54 studies in the qualitative synthesis. The included studies comprised 21 clinical studies, including observational cohorts and early non-randomized microbiota-intervention studies, 19 animal studies and 14 *in vitro* mechanistic studies, giving a total of 54 included studies. An aggregate number of unique clinical participants is not reported because several publications drew on overlapping or potentially overlapping patient datasets, preventing reliable de-duplication across studies. Study-specific sample sizes are therefore presented in **Supplementary Table S1** rather than summed across the clinical evidence base. **Supplementary Table S1** also provides the full citations and study-level characteristics of all 54 included studies, including studies contributing to the evidence map but not discussed individually in the narrative synthesis.

**Figure 3:**
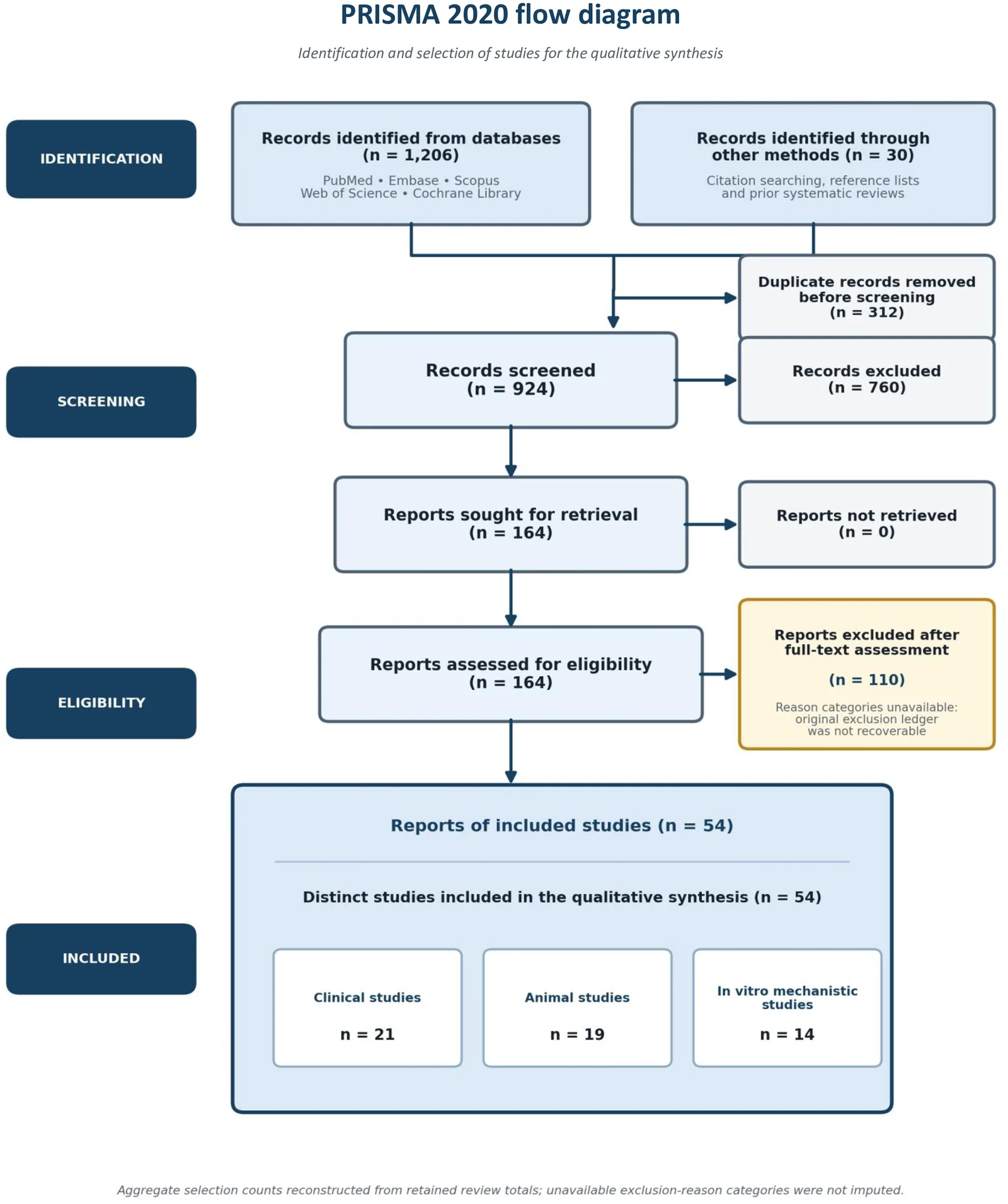
PRISMA 2020 flow diagram of study identification, screening, eligibility assessment and inclusion. Identification-stage and title-and-abstract screening counts were recovered from retained aggregate review records. During revision, the full-text selection ledger was reconstructed from retained review materials. The reconstructed ledger reproduced the original full-text eligibility decisions and exclusion categories. A second reviewer independently verified the reconstructed ledger against all 164 full-text reports, including the eligibility decision and primary exclusion reason assigned to each excluded report; disagreements were resolved by consensus, with third-reviewer adjudication where required. The mutually exclusive exclusion categories shown in the diagram total 110 reports. Original reviewer-specific title-and-abstract screening forms and database-export files were unavailable.

Several reports were examined closely because their subject matter appeared potentially compatible with the review questions but did not satisfy the final eligibility criteria. Petrelli et al. was excluded because it was a state-of-the-art review rather than a primary research study (47). The preliminary conference report by Derosa et al. was excluded as a secondary report of the same investigation because the corresponding full-length peer-reviewed publication was retained as the report of record (48). Clear et al. was excluded because it was available only as a conference abstract, without an identified full-length peer-reviewed report. Each report was assigned one mutually exclusive primary reason for exclusion in the reconstructed full-text ledger.

Each publication was counted once and classified according to its principal experimental design. The in vitro mechanistic studies were included to examine molecular and cellular pathways relevant to the microbiome–nitric oxide–immune checkpoint inhibitor axis. Their findings were considered separately from evidence of clinical benefit or therapeutic efficacy in vivo.

Most of the clinical literature concerned melanoma and non-small-cell lung cancer. Smaller numbers of studies included gastrointestinal, hepatobiliary, prostate, renal, and mixed solid tumors. The immune checkpoint inhibitors evaluated targeted PD-1, PD-L1, or CTLA-4 and were administered either as monotherapy or in combination regimens. PD-1 inhibitors included nivolumab and pembrolizumab; PD-L1 inhibitors included atezolizumab and durvalumab; and ipilimumab was the principal CTLA-4 inhibitor examined.

**Supplementary Table S1** presents the characteristics of the included studies, including study design, clinical population or experimental model, microbiome or nitric oxide exposure, immune checkpoint inhibitor regimen where applicable, analytical methods, principal outcomes, and assigned evidence domain. The identification and screening of records, full-text eligibility assessment, and final classification of the included studies are shown in the PRISMA 2020 flow diagram in **Figure 3**.

### Methodological quality and risk of bias

Among the 18 observational clinical studies assessed with the Newcastle–Ottawa Scale, five were judged to be of high methodological quality, eleven of moderate quality and two of low quality. The three non-randomized microbiota-intervention studies assessed separately with ROBINS-I were considered to be at serious risk of bias. No animal study met the conservative threshold for a low overall risk of bias: seventeen were judged to have an unclear overall risk and two a high overall risk. The principal concerns were incomplete reporting of sequence generation, allocation concealment, random housing and blinded outcome assessment.

Among the in vitro mechanistic studies, six satisfied most of the defined methodological-quality criteria, seven showed some limitations, and one had important limitations. These assessments were used to judge the relative weight of individual findings within the thematic synthesis rather than as grounds for automatic exclusion. Study-level judgments and the rationale supporting each assessment are presented in **Supplementary Table S2**.

### Domain 1 *–* microbial taxa associated with ICI response

Within the studies included in this review, enrichment of *Akkermansia muciniphila*, *Faecalibacterium prausnitzii*, *Bifidobacterium longum* and several other taxa was associated with favorable ICI outcomes in selected clinical cohorts and experimental models.

However, the reported microbial signatures were cohort-dependent and should not be interpreted as a comprehensive or universally reproducible ranking of microbial predictors.

#### Clinical evidence

Clinical cohort studies have linked gut-microbiome composition with outcomes following PD-1 or PD-L1 blockade, although the microbial signatures reported have varied across populations and analytical platforms. In patients with metastatic melanoma, Matson et al. found enrichment of Bifidobacterium longum, Collinsella aerofaciens, and Enterococcus faecium among responders (45). Routy et al. reported poorer outcomes following PD-1 blockade in patients with epithelial tumors who had been exposed to antibiotics or had a lower abundance of Akkermansia muciniphila (24).

The accompanying preclinical experiments provided a different level of evidence. Fecal microbiota transplantation (FMT) from responders improved tumor control and anti-PD-L1 activity in recipient mice, while administration of A. muciniphila restored sensitivity to PD-1 blockade in antibiotic-treated or germ-free mice (24, 45). The clinical observations remain associative. By contrast, the FMT and supplementation experiments provide causal support within the corresponding animal models, but not beyond them.

Additional prospective clinical evidence was reported in a cohort of 338 patients with advanced non-small-cell lung cancer, in which baseline fecal A. muciniphila was associated with objective response and overall survival independently of PD-L1 expression (48). Consistent with this, increased Faecalibacterium prausnitzii levels were observed in multiple melanoma and NSCLC cohorts, correlating with prolonged progression-free survival (PFS) by 4–9 months.

Systematic review evidence further identifies F. prausnitzii among the most consistently reported taxa associated with favorable ICI outcomes across cancer types (49).

However, despite these recurring associations, cohort analyses reveal limited reproducibility of microbiome signatures. Species such as Bifidobacterium pseudocatenulatum, Roseburia spp., and A. muciniphila have been variably associated with responders, suggesting that no single microbial species serves as a universal biomarker of ICI responsiveness (50, 51). This variability underscores the complexity of host–microbiota–immune interactions and highlights the need for standardized analytic frameworks in microbiome–immunotherapy research.

#### Preclinical and mechanistic evidence

Preclinical models have been instrumental in elucidating how specific microbial taxa modulate the efficacy of ICIs. Antibiotic-depleted or germ-free mice exhibit markedly diminished anti-PD-1 responses, underscoring the dependence of effective immunotherapy on an intact and immunocompetent gut microbiome. Building on this foundational understanding, recent evidence indicates that A. muciniphila actively remodels the tumor microenvironment (TME) to favor antitumor immunity through multiple complementary mechanisms.

The reported effects of Akkermansia muciniphila were tumor- and model-specific. In an HCC mouse model, supplementation increased intratumoral CD8^+^ T-cell proportions, reduced tumor-cell PD-L1 expression and enhanced anti-PD-1 activity (52). In gastric-cancer models, live A. muciniphila or its outer-membrane preparation promoted M1-like macrophage polarization, inflammatory cytokine expression and immune-cell infiltration, although checkpoint-blockade efficacy was not directly tested (53). In colorectal-cancer models, A. muciniphila induced TLR2/NLRP3-dependent M1-like tumor-associated macrophages (139). These findings support preclinical immunomodulatory activity but do not establish a common mechanism or clinical efficacy across tumor types.

Separately, preliminary data presented in a conference abstract suggested that dietary patterns associated increased A. muciniphila abundance may enhance the response to PD-L1 blockade in a preclinical model of triple-negative breast cancer (54). As no corresponding full-length, peer-reviewed publication was identified, the abstract was treated as contextual material and excluded from the formal evidence synthesis, study counts, quality appraisal and conclusions of this review.

These macrophage findings suggest a possible, but unproven, intersection with nitric oxide biology. The cited A. muciniphila studies did not directly demonstrate altered intratumoral iNOS activity or NO flux. Separate experimental studies indicate that iNOS-derived reactive nitrogen species may contribute to tumor-cell killing or restrain inflammatory macrophage programs through negative-feedback mechanisms, depending on cellular and redox context (55–58). A direct A. muciniphila–iNOS–ICI pathway therefore remains unproven. Separately, fecal microbiota transfer from ICI-responsive patients conferred improved tumor control in otherwise non-responsive germ-free mice, providing experimental support for a causal microbiome–immune relationship (45). Building on this microbiome-immune axis, the gut’s influence extends to systemic inflammation and vascular function, where nitric oxide (NO) is a key mediator (59). However, in inflammatory states like colitis, conventional NO markers such as nitrite and nitrate become unstable and dissociate from disease activity, revealing a complex metabolic picture that may be directly influenced by both microbial ecology and therapeutic agents like 5-ASA (59).

Experimental studies have linked A. muciniphila with dendritic-cell/IL-12-dependent T-cell responses in selected ICI models (24), whereas Ruminococcaceae-associated taxa have been linked more broadly with SCFA production, mucosal ecology and favorable ICI-associated microbial signatures (45). These observations arise from distinct experimental and clinical evidence streams and should not be interpreted as demonstrating a common mechanism. These effects are further amplified by microbial-derived metabolites such as short-chain fatty acids (SCFAs)—notably butyrate and propionate—which enhance memory CD8^+^ T-cell differentiation and support interferon-γ–mediated tumor cytotoxicity. Acting through G-protein–coupled receptor activation and histone deacetylase inhibition, SCFAs also enhance cytotoxic CD8^+^ T-cell activity while restraining regulatory T-cell expansion. Additionally, butyrate and propionate modulate endothelial nitric oxide synthase (eNOS)–dependent pathways, promoting vascular normalization and facilitating immune cell infiltration into tumors (60–62).

In addition, tryptophan metabolites derived from commensal species modulate aryl hydrocarbon receptor (AhR) signaling, influencing T-cell activation and tumor immune tolerance (63). Collectively, these preclinical and mechanistic data establish a biologically plausible framework linking gut microbiota composition, nitric oxide–associated immune modulation, mucosal priming, and systemic antitumor immunity. They also reinforce clinical observations that specific commensals such as A. muciniphila and F. prausnitzii act as immunomodulatory keystone taxa influencing ICI efficacy in a cohort-dependent manner.

### Domain 2 *–* selected microbial taxa and metabolites: context-dependent immune effects and hypothesized vascular links

The evidence concerning Akkermansia muciniphila and Ruminococcus-associated taxa derives from separate observational and experimental literatures rather than studies of a defined microbial consortium. In murine tumor models, live A. muciniphila enhanced anti-PD-1 activity by promoting cytotoxic CD8^+^ T-cell states and modifying immune interactions within the tumor microenvironment (29). Review-level literature has separately discussed selected Ruminococcus taxa in cancer-related settings (64), broader microbiome–immune and microbiome–ICI interactions (65, 68), and the immunological effects of A. muciniphila (66). However, no included primary study directly tested an A. muciniphila–Ruminococcus combination or demonstrated synergy between these taxa. Moreover, human melanoma studies associated ICI outcomes with the family Ruminococcaceae (25); this finding should not be interpreted as a uniform effect of the genus Ruminococcus.

The SCFA evidence was similarly dependent on metabolite, experimental model and treatment context. In primary experimental studies, pentanoate and butyrate enhanced cytotoxic T-lymphocyte and CAR-T-cell activity through metabolic and epigenetic reprogramming (74), while butyrate enhanced anti-PD-1 activity and T-cell-receptor signaling in cytotoxic CD8^+^ T cells in another experimental system (77). Reviews have considered the possible relevance of SCFAs and Clostridium butyricum CBM588 to checkpoint-inhibitor therapy (73) and summarized broader SCFA effects on CD4^+^ and CD8^+^ T-cell biology (78). These findings support context-dependent immune modulation but do not establish that Ruminococcus species uniformly produce a therapeutically beneficial SCFA profile.

The proposed vascular connection remains indirect. General literature on tumor metabolism, therapy-induced remodeling of the tumor microenvironment, and NO/S-nitrosylation demonstrates that NO can regulate endothelial adhesion and vascular biology in a context-dependent manner (42, 71, 75). These sources do not demonstrate that Ruminococcus-derived butyrate activates eNOS, normalizes tumor vasculature or increases immune-cell trafficking during ICI therapy. Similarly, acetate inhibited histone deacetylases and increased histone acetylation in primary CD4^+^ T cells in an HIV-1 model (72), but that non-oncology study cannot establish antitumor, vascular or checkpoint-inhibitor effects.

Several preliminary conference reports described tumor-associated changes in the gut microbiota (67), butyrate combined with an oncolytic adenovirus (69), butyrate in relation to CAR-T-cell therapy (70), and microbiome-associated immune activation during PD-1 blockade (76). Because corresponding full-length peer-reviewed publications were not identified, these reports were cited for contextual completeness only and were excluded from the formal evidence synthesis, study counts, methodological appraisal and conclusions of this review.

Taken together, the available evidence supports separate and context-dependent microbial, metabolite, immune and vascular pathways. It does not establish an A. muciniphila–Ruminococcus synergy or a causal Ruminococcus–butyrate–eNOS–vascular-normalization–ICI pathway.

### Domain 3 *–* nitric oxide signaling in antitumor immunity

Nitric oxide is a context-dependent regulator of tumor immunity rather than a molecule with a simple concentration-dependent division between beneficial and harmful effects. Its biological consequences are jointly determined by cellular source, local flux, duration and spatial distribution of exposure, tumor stage, oxygen tension and the surrounding redox environment (79–82). Accordingly, similar nominal concentrations may produce different effects in different experimental or tissue contexts.

In selected experimental systems, controlled NO exposure has enhanced dendritic-cell recruitment, cytotoxic T-cell infiltration, inflammatory cytokine production or tumor-cell killing (83, 84). In other settings, sustained or spatially restricted NO production—particularly from tumor-associated macrophages or myeloid-derived suppressor cells—has impaired T-cell receptor signaling, promoted immunosuppressive phenotypes and supported tumor progression or treatment resistance (85–87). These apparently divergent findings reflect differences in source, timing, cellular compartment and redox context rather than a universally applicable low-versus-high concentration rule.

Clinical translation therefore requires pathway- and context-specific modulation. NO augmentation may be beneficial in experimental settings characterized by deficient vascular or cytotoxic signaling, whereas inhibition may be appropriate when sustained iNOS-related activity contributes to immune suppression. Current evidence does not establish a universally effective direction or magnitude of NO modulation for patients receiving ICIs.

From a translational standpoint, this bidirectionality presents both therapeutic promise and caution for integrative oncology. In preclinical models, low-dose NO donors have demonstrated immunomodulatory antitumor activity, including increased tumor-infiltrating CD8^+^ T cells and dendritic cells, and their combination with cisplatin produced additive tumor-suppressive effects (84). These findings support the biological plausibility of NO-mediated modulation of antitumor immunity but do not establish synergy between NO modulation and immune checkpoint blockade. Separately, inhibition of iNOS within the tumor microenvironment has been reported to enhance responsiveness to TLR agonists and radiotherapy by alleviating NO-associated immunosuppression (88). Effective clinical translation therefore requires precise, context-dependent modulation, balancing NO’s immune-activating potential against its capacity to support immunosuppression or tumor progression.

#### Domain 3.1 *–* microbial and host-derived NO pathways supporting antitumor immunity

Across the studies included in this Qualitative Systematic Review, three mechanistic patterns consistently emerged. First, an enzymatic, NOS-dependent pathway was highlighted in several preclinical studies, which demonstrated that moderate iNOS upregulation within tumor-associated macrophages (TAMs) was associated with enhanced cytotoxic function (54). This is supported by evidence of iNOS-mediated tumor cell destruction, where iNOS-expressing cells achieved near-complete killing of ovarian cancer cells (89). High-output nitric oxide generated by pro-inflammatory (M1) macrophages via iNOS can induce tumor cell apoptosis and necrosis through DNA damage, p53 stabilization, and mitochondrial dysfunction, supporting early antitumor immune surveillance (90). Consistently, iNOS knockout models demonstrate increased susceptibility to tumor initiation, supporting a tumor-suppressive role for high-output NO in early immune surveillance (91).

However, NO signaling is strongly context dependent. Its biological effects vary with cellular source, local flux, exposure duration, spatial compartment, oxygen tension, tumor model, redox environment, and measurement method. The included studies used heterogeneous NO-generating systems, donors, experimental models, and analytical approaches; they therefore do not support a universal quantitative threshold or therapeutic window. Antitumor and protumor effects have both been observed across experimental systems, and concentration alone is insufficient to predict biological outcome. In established hypoxic tumors, changes in macrophage phenotype, oxygen availability, NOS activity, and local redox conditions may shift NO-associated immune and vascular effects, but these transitions cannot be defined by a single concentration range (44, 91–93).

Second, the included literature documents oral microbial nitrate reduction and enterosalivary conversion of nitrate to nitrite, processes that contribute to systemic nitrate and nitrite handling and may support subsequent NO generation. Oral nitrate-reducing taxa, including Veillonella, Neisseria, and Actinomyces species, participate in this physiology (38, 94–97). These observations establish systemic biological plausibility but not a tumor-specific mechanism. No included ICI study demonstrated that microbial nitrate reduction maintains intratumoral NO during tumor hypoxia, improves tumor oxygenation, modifies immune-cell infiltration, or changes treatment response. The proposed connection between microbial nitrate metabolism and intratumoral NO biology therefore remains indirect and hypothesis-generating.

Third, a peer-reviewed murine study offers model-specific proof of concept that localized intratumoral delivery of high-concentration gaseous NO can modify the activity of PD-1 blockade in the CT26 model (98). This intervention differs both pharmacologically and in its site of delivery from physiological NOS-derived NO and microbiota-supported nitrate–nitrite metabolism.

Confino et al. studied immunocompetent BALB/c mice bearing bilateral CT26 colon tumors and administered intratumoral gaseous NO at 50,000 ppm for either 5 or 10 minutes, together with systemic anti-mPD-1 treatment (98). Nine days after NO administration, the 10-minute combination regimen resulted in a significantly smaller primary-tumor volume than anti-mPD-1 alone (p = 0.0005). By day 100, 53% of mice in the combination group remained free of both primary and secondary tumors. The difference in tumor-free status between groups was not statistically significant, however (p = 0.1489), and neither was the survival comparison (p = 0.0653).

These results support further investigation of localized high-concentration NO in preclinical ICI models. They do not demonstrate clinical efficacy, establish generalizability across tumor types, or provide evidence of a causal link with dietary or microbiota-derived NO.

### Domain 4 *–* gut microbiota, vascular normalization, and immune infiltration evidence: microbial signatures linked to vascular phenotype and immune access

Across both clinical and preclinical studies, Akkermansia muciniphila has emerged as one of the most consistently enriched microbial taxa in patients responding to immune checkpoint inhibitors (ICIs). In non–small cell lung cancer (NSCLC), enrichment of A. muciniphila reliably distinguishes responders and is associated with enhanced CD8^+^ T-cell infiltration and more activated cytokine signatures within the tumor microenvironment (24, 47, 99). Routy et al. first demonstrated that the presence of A. muciniphila restored anti–PD-1 efficacy in antibiotic-treated or germ-free mouse models by increasing recruitment of CCR9^+^CXCR3^+^CD4^+^ and CD8^+^ T cells to tumors (24). Subsequent clinical analyses by Derosa et al. reinforced these findings, showing that A. muciniphila–positive NSCLC patients exhibited superior objective response rates and improved overall survival compared with those lacking this taxon (47, 99).

Experimental administration of A. muciniphila in murine models further increases intratumoral CD8^+^ T cells and enhances antitumor activity, suggesting improved immune access to the tumor bed (29).

Short-chain fatty acid (SCFA)–producing taxa have shown similar relevance, as butyrate and propionate support the effector functions of CD8^+^ T cells and NK cells in vivo (100). Preclinical vascular biology studies, although not conducted in cancer models, show that SCFAs modulate endothelial barrier integrity, regulate junctional protein expression, and alter cytokine-induced adhesion molecule dynamics (VCAM-1 and ICAM-1) (100–102). These findings provide a plausible mechanism through which microbial metabolites may influence immune cell extravasation into tumors.

### Mechanism: microbial metabolites and nitric oxide in vascular modulation

Studies in cultured endothelial cells and non-oncology preclinical models indicate that SCFAs can influence G-protein-coupled receptor signaling, HDAC activity, inflammatory cytokine production, endothelial adhesion-molecule expression, and barrier function (24, 99–102). A separate body of vascular research shows that host eNOS-derived NO contributes to endothelial homeostasis and vascular regulation. Considered together, these findings offer indirect mechanistic support for the possibility that microbial metabolites and NO-related signaling shape vascular phenotypes relevant to immune-cell trafficking. The cited studies do not, however, show that microbiota-supported nitrate reduction normalizes tumor vasculature, improves tumor perfusion, or increases leukocyte entry during ICI treatment. Direct evidence of functional interaction between SCFAs and NO within tumor vessels remains lacking.

### Synthesis: an integrative model of microbiome-driven immune access

Clinical and preclinical studies have linked microbiome features with ICI outcomes and tumor-immune phenotypes (24, 25, 45, 48, 50). A separate body of endothelial research shows that microbial metabolites, particularly SCFAs, can affect endothelial barrier function, adhesion-molecule expression, and inflammatory signaling (24, 99–103). Tumor-vascular studies further indicate that vascular dysfunction can reduce perfusion and limit immune-cell entry into tumors (104–106).

Whether these findings converge through microbiome-driven normalization of tumor vasculature during ICI therapy remains uncertain. None of the studies included in this review demonstrated that modification of a gut microbial taxon converts an immune-excluded tumor into an immune-inflamed phenotype by normalizing the vasculature. This mechanism therefore remains an integrative hypothesis that requires direct experimental testing.

### Translational input: therapeutic opportunities and future directions

The mechanistic insights emerging from this domain reveal several translational opportunities for enhancing ICI efficacy through targeted manipulation of the microbiome–vascular–immune axis. Approaches that increase the availability of microbially derived metabolites, such as the use of targeted prebiotics to expand SCFA-producing taxa or the administration of purified postbiotics, may help promote endothelial stabilization and improve immune cell access to tumors (107). Precision microbiome modulation, including supplementation with A. muciniphila or defined SCFA-producing microbial consortia currently under investigation, offers a complementary strategy for augmenting ICI responsiveness through vascular and immunologic conditioning (108, 109). Emerging mechanistic evidence suggests that pharmacologic strategies capable of mimicking microbially derived metabolite signaling, particularly SCFA-mediated HDAC inhibition and microbiome-supported nitrate–nitrite–NO pathways, may offer a feasible means of enhancing tumor vascular normalization and immune accessibility (61, 110, 111). Histone deacetylase (HDAC) inhibitors are of particular interest because they reproduce several downstream cellular effects attributed to butyrate, including HDAC inhibition, chromatin remodeling, and transcriptional reprogramming of endothelial and immune cells (112–115). These actions parallel butyrate’s reported ability to reduce inflammatory cytokine production, stabilize endothelial junctions, and modulate adhesion molecule expression, thereby creating conditions favorable for immune cell trafficking (100, 116). Specifically, butyrate suppresses TNF-α–induced expression of the vascular adhesion molecules VCAM-1 and ICAM-1 in endothelial cells, inhibits NF-κB activation to reduce pro-inflammatory cytokine production, and decreases monocyte adhesion to the endothelium (101, 116–118), collectively supporting a more regulated and less inflammatory vascular environment. Separately, experimental and non-oncology vascular studies provide a rationale for investigating whether modulation of NO bioavailability influences endothelial and perfusion phenotypes relevant to tumor immunity (84, 119). These data do not establish that nitrate/nitrite administration or other NO-directed interventions normalize tumor vasculature or enhance ICI efficacy. Imaging measures of perfusion, vascular permeability and hypoxia could therefore serve as exploratory pharmacodynamic endpoints in future studies testing this hypothesis (120–123), rather than as established biomarkers of a therapeutically favorable microbiome–NO state.

### Domain 5 *–* modulation of the nitrate*–*nitrite*–*NO pathway to enhance ICI response

Across the included literature, microbiota- and nitrate-related pathways offered a mechanistic basis for examining whether altered NO bioavailability could influence vascular and immune phenotypes relevant to ICI response. Most of this evidence comes from animal models, cellular studies, and human pharmacokinetic or vascular investigations conducted outside oncology. The findings support biological plausibility, but they do not show that dietary nitrate, nitrate-reducing microorganisms, or other NO-directed interventions improve responses to immune checkpoint inhibitors in patients with cancer.

### Microbiota-mediated modulation

Responder-derived microbiota have shown biological effects in preclinical transfer models and early clinical signals in small, non-randomized studies. In germ-free or antibiotic-treated mice, microbiota transferred from ICI responders improved tumor control and enhanced the effects of PD-1 or PD-L1 blockade, whereas microbiota from non-responders produced no comparable benefit (24, 25, 45). These experiments provide causal evidence within the models studied, but they do not establish efficacy in patients.

Baruch et al. evaluated responder-derived fecal microbiota transplantation (FMT) combined with reinduction of anti-PD-1 therapy in a phase I study involving 10 patients with anti-PD-1-refractory metastatic melanoma (124). Three patients responded: two achieved partial responses and one achieved a complete response. In a separate small, non-randomized study of 13 patients with anti-PD-1-refractory advanced solid cancers, Kim et al. reported clinical benefit in six patients, including one partial response and five cases of stable disease (125).

These studies provide preliminary proof of concept in humans, but the findings remain difficult to interpret. The cohorts were small, donors and recipients were heterogeneous, anti-PD-1 therapy was administered concurrently, and neither study included a randomized comparator group. It also remains unclear which microbial organisms or functions accounted for the observed outcomes (124, 125). Contemporary reviews therefore continue to regard FMT and other microbiome-directed interventions as investigational approaches requiring validation in controlled clinical studies (126, 127).

None of these studies showed that nitrate–nitrite–NO signaling mediated the preclinical or clinical effects of FMT (24, 25, 45, 124, 125). Any proposed link between responder-derived microbiota, NO biology, and ICI responsiveness should therefore be treated as a hypothesis for direct testing rather than as an established mechanism.

### Dietary nitrate/nitrite supplementation

Oral inorganic nitrate increases circulating and salivary nitrate/nitrite and can modify selected vascular endpoints in non-oncology human studies (129–133). These observations establish systemic pharmacokinetic and physiological activity but do not demonstrate intratumoral NO delivery, tumor-vessel normalization, immune-cell infiltration or improved ICI efficacy. The available oncology evidence is limited to indirect animal or cellular models (134–136). No clinical study included in this review evaluated nitrate or nitrite as an adjunct to ICI therapy. Human studies consistently demonstrate that dietary nitrate (standardized for nitrate)—typically delivered through beetroot juice or inorganic nitrate salts—reliably increases circulating nitrate and nitrite levels, confirming effective bioconversion along the nitrate–nitrite–NO pathway (128, 129). The elevation in plasma nitrite has been shown to be dose-dependent, with peak increases occurring 2–3 hours after ingestion (128). These pharmacokinetic findings are reinforced by evidence that dietary nitrate augments systemic nitrate stores and enhances circulating nitrite levels (129). Across multiple trials, participants have exhibited 3–7-fold rises in plasma and salivary nitrate/nitrite concentrations within hours of intake (130), changes which occur in parallel with measurable improvements in endothelial and macrovascular function (131–133). However, the translational effectiveness of this strategy is contingent on an intact nitrate–nitrite–NO pathway; in many patients, underlying gut and oral dysbiosis (from mouthwash use, antibiotics or other antiseptics such as fluoride) may impair microbial nitrate reduction, limiting nitrite generation and downstream NO signaling despite adequate nitrate intake.

Preclinical oncology models provide mechanistic rationale for testing nitrate interventions in the context of cancer therapy. Dietary nitrate has demonstrated protective effects against radiation-induced tissue injury by maintaining normal tissue function and perfusion. Xiaoyu Feng et al. showed that nitrate prevented radiation-induced xerostomia in miniature pigs by preserving parotid gland function (134). D. Citrin et al. note that few radioprotective agents are clinically approved, but emerging compounds such as dietary nitrate show promising preclinical results (135). These findings provide indirect evidence that nitrate–nitrite–NO biology can influence tissue perfusion, hypoxia and radiation-related injury. However, radioprotection models do not establish that dietary nitrate improves antitumor immunity or ICI efficacy, and their relevance to checkpoint blockade remains hypothesis-generating. Notably, nitrite signaling exhibits a biphasic, stage-dependent profile—suppressing proliferation and invasion in early-stage colon cancer cells while promoting progression in advanced-stage disease at higher concentrations (136).

Despite this mechanistic promise, direct clinical evidence for dietary nitrate or nitrite as an adjunct to ICIs is currently lacking. While cardiovascular and metabolic trials establish biological plausibility (increased plasma nitrite/nitrate and improved endothelial function), no completed randomized oncology trials to date have demonstrated that nitrate supplementation improves ICI response rates or survival (126, 127). Ongoing and future early-phase studies should therefore prioritize integrated biomarker endpoints to test the mechanistic hypothesis rather than rely solely on clinical response as a primary outcome (126, 127).

Dietary nitrate or nitrite supplementation can increase systemic nitrate and nitrite availability, and preclinical studies provide indirect evidence that nitrate–nitrite–NO biology may influence vascular and hypoxic phenotypes relevant to tumor immunity. Oncology-specific evidence remains limited, however, and no clinical study identified in this review demonstrated improved ICI response, progression-free survival or overall survival. Prospective studies incorporating pharmacokinetic, vascular, immune and microbiome biomarkers are required to determine whether this mechanistic hypothesis has therapeutic relevance in patients receiving immune checkpoint inhibitors.

### Potential interaction between dietary nitrate and microbial pathways

Dietary nitrate and Akkermansia muciniphila have each been associated with vascular or immune pathways that may be relevant to antitumor immunity, but the evidence arises from separate experimental literatures. Dietary nitrate supports nitrate–nitrite–NO metabolism and has demonstrated pharmacokinetic and vascular effects predominantly in non-oncology human studies and indirect preclinical models (43, 135–137). In experimental systems, A. muciniphila has shown immunomodulatory activity, including induction of TLR2/NLRP3-mediated M1-like tumor-associated macrophages and enhancement of IL-2-based immunotherapy (138–140).

No included study directly evaluated dietary nitrate and A. muciniphila in combination. The available evidence therefore does not establish additive or synergistic effects on tumor vascular normalization, endothelial function, immune-cell infiltration, ICI response or survival. Combined modulation of microbial composition and nitrate–nitrite–NO biology should be regarded as a mechanistic hypothesis requiring direct evaluation in appropriately controlled preclinical studies before clinical testing.

### Summary of emerging patterns

Across the evidence base, several themes recur:

- A. muciniphila is one of the most consistent microbiome signatures associated with ICI responsiveness.
- Enzymatic and microbiota-associated NO pathways may converge on vascular and immune processes relevant to perfusion, immune-cell trafficking and T-cell infiltration, although direct evidence linking this convergence to improved ICI outcomes remains limited.
- Integrated modulation of microbial composition and NO-related pathways is biologically plausible, but its safety, optimal implementation and clinical efficacy in ICI-treated patients remain unestablished.

Overall, Domain 5 provides a biological basis for examining whether microbiota-directed interventions, combined with dietary or metabolic modulation of NO pathways, might influence antitumor immunity and responsiveness to ICIs. The efficacy and safety of this strategy, including any additive effects, have not yet been established and will require direct evaluation in controlled preclinical studies and prospective clinical trials.

### Limitations

This review was designed to address a defined mechanistic question, not to catalog every microbiome correlate reported in association with ICI response. Although the search included broad microbiome and immune-checkpoint terminology, its objectives and search framework gave particular attention to Akkermansia muciniphila, Ruminococcus spp., microbial metabolites, nitrate–nitrite–NO biology, and tumor–vascular pathways. The prominence of individual taxa in this synthesis should therefore not be taken as an unbiased measure of their prevalence, predictive value, or importance relative to taxa lying outside the review’s mechanistic scope. This targeted approach carries a risk of scope-related selection and confirmation bias and limits the extent to which the findings can be generalized to the broader microbiome-biomarker literature.

Third, the context-dependent biology of NO remains a major source of translational uncertainty. The biological effects of NO vary with cellular source, local flux, exposure duration, spatial compartment, oxygen tension, tumor model, cellular composition, redox environment, and measurement method. These variables differed substantially among the included studies, preventing the inference of a universal quantitative threshold or therapeutic window. Systemic nitrite or nitrate concentrations also cannot be assumed to reflect bioactive NO within the tumor microenvironment. Clinically meaningful concentration–response relationships will require spatially and temporally resolved measurements in human tumors, together with simultaneous assessment of immune, vascular, and tumor-cell responses. Until such data are available, NO-modulating interventions cannot be dosed or selected on the basis of a single proposed concentration range. Miscalibrated NO could counterproductively promote angiogenesis or immunosuppression.

Finally, microbiome function is inherently ecological and personalized. Focusing on single taxa such as Akkermansia muciniphila risks oversimplification, as therapeutic benefits depend on community-level interactions, substrate availability, and host-specific factors. Most clinical datasets originate from Western populations, limiting generalizability, and interventional studies directly testing NO-modulating or nitrate-reducing microbial strategies in humans are scarce. The review was not prospectively registered, and no protocol was published before study screening and data extraction began. It is therefore not possible for readers to determine independently whether all methodological decisions were specified in advance. In particular, the evidence-stratum eligibility criteria were clarified during revision; this retrospective formalization may have influenced study classification and should be considered when interpreting the synthesis. To mitigate this limitation, we report the search strategies, eligibility criteria, study-selection procedures, evidence-classification framework, risk-of-bias assessment, and reasons for full-text exclusion in detail. Despite these limitations, convergence across molecular, vascular, and immunologic data supports the biological plausibility of the microbiome–NO axis and underscores its potential as a future therapeutic target.

### Future directions: a translational roadmap for the microbiome*–*NO*–*ICI axis

Advancing the microbiome–nitric oxide–immunotherapy axis from conceptual framework to clinical utility requires a coordinated, mechanistically grounded research agenda spanning standardization, causality, biomarker development, and therapeutic innovation.

#### 1 Standardization and multi-omic precision

Meaningful progress depends on harmonizing analytic methods for both microbiome and NO biology. Future studies should adopt standardized shotgun metagenomics, consistent nitrite/nitrate and S-nitrosothiol panels, and direct TME-level NO assessment. Integrating metagenomics with metatranscriptomics, metabolomics (SCFAs, tryptophan metabolites, NO species), and deep tumor immunophenotyping will enable functional mapping of microbial pathways to vascular and immune phenotypes, shifting the field from taxonomic associations to mechanistic signatures.

#### 2 Establishing causality through precision microbial manipulation

To move beyond observational correlations, causality must be tested with higher-resolution models. Approaches include gnotobiotic mice colonized with defined consortia differing only in nitrate-reduction or SCFA-production capacity; CRISPR or phage-based tools to edit microbial functions in situ; and isogenic strain comparisons to identify gene clusters responsible for immunoregulatory activity. These systems will clarify whether key taxa such as Akkermansia and Ruminococcus act as true effectors or proxies of broader ecological states.

#### 3 Quantifying the axis: biomarkers and imaging-based readouts

Next-generation biomarkers should capture function, not just taxonomy. Fecal and plasma assays quantifying microbial nitrate-reduction potential, SCFA profiles, and systemic NO flux should be paired with dynamic imaging. Techniques such as dynamic contrast-enhanced MRI for perfusion and PET hypoxia tracers such as ¹^8^F-FMISO could serve as exploratory pharmacodynamic measures in studies testing the proposed microbiome–NO–vascular relationship. Changes in these measures should not be interpreted as evidence of an active microbiome–NO axis unless they are prospectively linked to microbial function, intratumoral NO biology, vascular phenotype, and immune-cell trafficking. These modalities are well suited for early pharmacodynamic endpoints in interventional trials.

#### 4 Rational, mechanistically driven clinical trials

Clinical translation will require studies built around clearly defined mechanisms, appropriate safety monitoring and functional biomarkers. To illustrate potential research directions, two hypothetical study concepts are outlined below. The names are working labels only; they do not refer to registered trials, finalized protocols or interventions currently ready for clinical implementation.

- Illustrative nitrate–ICI study concept (working label: “NITRATE-ICI”): An initial dose-escalation and feasibility study could evaluate a chemically defined dietary-nitrate intervention as an adjunct to standard-of-care ICI therapy. Primary objectives would include safety, tolerability and biological target engagement, with monitoring of blood pressure, methemoglobin, renal function, concomitant vasodilator use, nitrate/nitrite pharmacokinetics and exploratory tumor-vascular or intratumoral NO-related biomarkers. Randomized efficacy testing would be appropriate only after acceptable safety and target engagement had been demonstrated.
- Illustrative FMT–ICI study concept (working label: “FMT-Response”): A prospective early-phase study could evaluate rigorously screened responder-derived FMT or a defined microbial consortium alongside standard anti-PD-1 therapy in a defined ICI-refractory population. Safety and feasibility should remain the primary outcomes, with microbial engraftment, metagenomic and metabolomic changes, immune-cell phenotypes and tumor-microenvironmental effects assessed as mechanistic outcomes. Antitumor activity should be considered exploratory until evaluated in an adequately controlled trial.

#### 5 Designing next-generation therapeutics targeting the axis

The field is poised to develop precision therapeutics purpose-built to modulate this axis. Candidates include defined microbial consortia combining immunostimulatory and metabolic functions; postbiotics such as stabilized SCFA or NO-releasing formulations; and pharmacologic mimetics (e.g., HDAC inhibitors, eNOS activators) that recapitulate microbial vascular-benefit pathways with predictable pharmacokinetics.

#### 6 Expanding global diversity and personalization

Finally, expanding cohorts to African, Asian, and South American populations is essential for uncovering distinct microbial metabolic capacities and improving global applicability. Given the profound inter-individual variability of the microbiome, the ultimate translational path is likely to be personalized—anchored in functional microbial capacities rather than universal “beneficial” taxa.

## Conclusion and recommendations

This qualitative systematic review identifies a biologically plausible, although still provisional, framework linking the gut microbiome, nitric oxide biology, and responses to immune checkpoint inhibition. Human cohort studies have associated several taxa—including Akkermansia muciniphila, Faecalibacterium prausnitzii, Bifidobacterium species, and other short-chain fatty acid–producing organisms—with favorable ICI outcomes. These associations have not been consistent across cohorts. Variation in population characteristics, cancer type, treatment exposure, sample collection, and microbiome-analysis methods also limits the reproducibility of individual microbial signatures.

Microbial-transfer experiments and other animal studies provide causal evidence that microbiome composition can influence antitumor immunity within the experimental models examined. Early non-randomized fecal microbiota transplantation (FMT) studies extend this observation to humans by offering preliminary proof of concept. The clinical evidence remains limited, as the available studies are too small and insufficiently controlled to establish efficacy, identify the patients most likely to benefit, or determine which microbial organisms or functions account for the observed effects.

Beyond these intervention studies, mechanistic evidence suggests that microbial metabolites, host nitric oxide synthase pathways, and reactive nitrogen species may alter immune-cell function, vascular regulation, tumor hypoxia, and immune-cell trafficking. Direct evidence linking microbiota-mediated nitrate reduction to ICI efficacy nevertheless remains limited and largely indirect. The biological effects of NO depend on its cellular source, local flux, exposure duration, spatial compartment, oxygen tension, tumor type, and redox context. The current evidence therefore does not support a universal quantitative threshold or a clinically applicable therapeutic window.

Human studies of dietary nitrate conducted outside oncology have demonstrated increases in circulating nitrate and nitrite, together with measurable vascular effects. No clinical evidence currently shows, however, that dietary nitrate or other NO-directed interventions improve treatment response or survival in patients receiving ICIs. Microbiota-targeted interventions likewise remain investigational and should not yet be considered established adjuncts to checkpoint blockade.

At present, the microbiome–NO–ICI axis is best viewed as a testable integrative model for guiding mechanistic and translational research, rather than as a clinically actionable therapeutic strategy. Further progress will depend on more standardized measurements of microbiome function and NO biology, prospective studies linking microbial activity to intratumoral vascular and immune phenotypes, and biomarker-driven clinical trials capable of establishing causality, safety, and therapeutic efficacy.

## Data Availability

The original contributions presented in the study are included in the article/**Supplementary Material**. Further inquiries can be directed to the corresponding author.
