# Supplementary material for: The microbiome–nitric oxide axis in cancer immunotherapy: a targeted qualitative systematic review of mechanistic and translational evidence
Source: Front Immunol. 2026 Sep 24;17:1904698. doi: 10.3389/fimmu.2026.1904698 (PMC13646043; doi:10.3389/fimmu.2026.1904698)
Supplement: Supplementary file 1 [file DataSheet1.docx]

Supplementary File S3. PRISMA 2020 checklist

**The Microbiome–Nitric Oxide Axis in Cancer Immunotherapy: A Targeted Qualitative Systematic Review of Mechanistic and Translational Evidence**

**Reconstruction note.** This checklist maps PRISMA 2020 items to the revised manuscript and supplementary files. The original record-level screening ledger and reviewer-specific appraisal forms were unavailable during revision. Accordingly, the study inventory and study-level appraisal were retrospectively reconstructed from the manuscript citation corpus and cited reports. Unavailable full-text exclusion reasons, exact historical database strings and reviewer-agreement statistics are disclosed as unavailable rather than recreated.

*The checklist documents reporting location and transparent disposition; it does not convert unavailable archival records into recovered data.*

# Part A. PRISMA 2020 main checklist

| **Section/topic** | **Item** | **PRISMA 2020 item** | **Location in revised report / transparent disposition** |
| --- | --- | --- | --- |
| Title | 1 | Identify the report as a systematic review. | Title |
| Abstract | 2 | See the PRISMA 2020 for Abstracts checklist. | Structured Abstract; see Part B of this checklist |
| Rationale | 3 | Describe the rationale for the review in the context of existing knowledge. | Introduction |
| Objectives | 4 | Provide an explicit statement of the objective(s) or question(s) the review addresses. | Introduction; Methods—Study Design |
| Eligibility criteria | 5 | Specify the inclusion and exclusion criteria for the review and how studies were grouped for the syntheses. | Methods—Eligibility Criteria and Evidence-Stratum Classification |
| Information sources | 6 | Specify all databases, registers, websites, organisations, reference lists and other sources searched or consulted to identify studies. Specify the date when each source was last searched or consulted. | Methods—Search Strategy. Historical database concepts and coverage are reported; exact archived database strings and source-specific final run dates were not recoverable. |
| Search strategy | 7 | Present the full search strategies for all databases, registers and websites, including any filters and limits used. | Methods—Search Strategy. Core Boolean concepts are reported; complete historical platform-specific search strings were not recoverable. |
| Selection process | 8 | Specify the methods used to decide whether a study met the inclusion criteria of the review, including how many reviewers screened each record and each report retrieved, whether they worked independently, and if applicable, details of automation tools used in the process. | Methods—Study Selection and Data Extraction; Limitations—Review-process reconstruction. Aggregate selection counts were retained, but the original record-level decisions were unavailable. |
| Data collection process | 9 | Specify the methods used to collect data from reports, including how many reviewers collected data from each report, whether they worked independently, any processes for obtaining or confirming data from study investigators, and if applicable, details of automation tools used in the process. | Methods—Study Selection and Data Extraction; Supplementary Table S1. The revision-stage evidence map was reconstructed from cited reports. |
| Data items | 10a | List and define all outcomes for which data were sought. Specify whether all results that were compatible with each outcome domain in each study were sought (e.g. for all measures, time points, analyses), and if not, the methods used to decide which results to collect. | Methods—Eligibility Criteria; Study Selection and Data Extraction |
| Data items | 10b | List and define all other variables for which data were sought (e.g. participant and intervention characteristics, funding sources). Describe any assumptions made about any missing or unclear information. | Methods—Study Selection and Data Extraction |
| Study risk of bias assessment | 11 | Specify the methods used to assess risk of bias in the included studies, including details of the tool(s) used, how many reviewers assessed each study and whether they worked independently, and if applicable, details of automation tools used in the process. | Methods—Quality and Risk-of-Bias Assessment; Supplementary Table S2; Limitations. Original reviewer forms were unavailable; study-level judgments were retrospectively reconstructed. |
| Effect measures | 12 | Specify for each outcome the effect measure(s) (e.g. risk ratio, mean difference) used in the synthesis or presentation of results. | Methods—Data Synthesis Approach; Results |
| Synthesis methods | 13a | Describe the processes used to decide which studies were eligible for each synthesis (e.g. tabulating the study intervention characteristics and comparing against the planned groups for each synthesis (item #5)). | Methods—Eligibility Criteria and Data Synthesis Approach |
| Synthesis methods | 13b | Describe any methods required to prepare the data for presentation or synthesis, such as handling of missing summary statistics, or data conversions. | Methods—Data Synthesis Approach |
| Synthesis methods | 13c | Describe any methods used to tabulate or visually display results of individual studies and syntheses. | Figures 1–3; Main Table 1; Supplementary Tables S1 and S2 |
| Synthesis methods | 13d | Describe any methods used to synthesize results and provide a rationale for the choice(s). If meta-analysis was performed, describe the model(s), method(s) to identify the presence and extent of statistical heterogeneity, and software package(s) used. | Methods—Data Synthesis Approach |
| Synthesis methods | 13e | Describe any methods used to explore possible causes of heterogeneity among study results (e.g. subgroup analysis, meta-regression). | Methods—Data Synthesis Approach; Limitations |
| Synthesis methods | 13f | Describe any sensitivity analyses conducted to assess robustness of the synthesized results. | Not conducted |
| Reporting bias assessment | 14 | Describe any methods used to assess risk of bias due to missing results in a synthesis (arising from reporting biases). | Methods—Data Synthesis / reporting-bias limitation. No formal reporting-bias assessment was undertaken. |
| Certainty assessment | 15 | Describe any methods used to assess certainty (or confidence) in the body of evidence for an outcome. | Methods—Data Synthesis / certainty limitation. No formal certainty-grading framework was applied. |
| Study selection | 16a | Describe the results of the search and selection process, from the number of records identified in the search to the number of studies included in the review, ideally using a flow diagram. | Results—Study Selection and Characteristics; Figure 3 |
| Study selection | 16b | Cite studies that might appear to meet the inclusion criteria, but which were excluded, and explain why they were excluded. | Figure 3; Limitations—Review-process reconstruction. The original report-level full-text exclusion reasons were unavailable and were not invented retrospectively. |
| Study characteristics | 17 | Cite each included study and present its characteristics. | Supplementary Table S1; Results |
| Risk of bias in studies | 18 | Present assessments of risk of bias for each included study. | Supplementary Table S2; Results—methodological appraisal; Limitations—original reviewer forms unavailable. |
| Results of individual studies | 19 | For all outcomes, present, for each study: (a) summary statistics for each group (where appropriate) and (b) an effect estimate and its precision (e.g. confidence/credible interval), ideally using structured tables or plots. | Results; Supplementary Table S1 |
| Results of syntheses | 20a | For each synthesis, briefly summarise the characteristics and risk of bias among contributing studies. | Results; Supplementary Tables S1 and S2 |
| Results of syntheses | 20b | Present results of all statistical syntheses conducted. If meta-analysis was done, present for each the summary estimate and its precision (e.g. confidence/credible interval) and measures of statistical heterogeneity. If comparing groups, describe the direction of the effect. | No statistical synthesis performed |
| Results of syntheses | 20c | Present results of all investigations of possible causes of heterogeneity among study results. | No formal heterogeneity investigation performed |
| Results of syntheses | 20d | Present results of all sensitivity analyses conducted to assess the robustness of the synthesized results. | No sensitivity analyses performed |
| Reporting biases | 21 | Present assessments of risk of bias due to missing results (arising from reporting biases) for each synthesis assessed. | Limitations. No formal reporting-bias assessment was undertaken. |
| Certainty of evidence | 22 | Present assessments of certainty (or confidence) in the body of evidence for each outcome assessed. | Limitations. No formal certainty-of-evidence grading was undertaken. |
| Discussion | 23a | Provide a general interpretation of the results in the context of other evidence. | Results and Discussion; Conclusion and Recommendations |
| Discussion | 23b | Discuss any limitations of the evidence included in the review. | Limitations |
| Discussion | 23c | Discuss any limitations of the review processes used. | Limitations—lack of prospective registration, unavailable record-level screening ledger, unavailable original reviewer forms and post hoc reconstruction. |
| Discussion | 23d | Discuss implications of the results for practice, policy, and future research. | Future Directions; Conclusion and Recommendations |
| Registration and protocol | 24a | Provide registration information for the review, including register name and registration number, or state that the review was not registered. | Methods—Protocol and Registration; Limitations |
| Registration and protocol | 24b | Indicate where the review protocol can be accessed, or state that a protocol was not prepared. | Methods—Protocol and Registration; Limitations |
| Registration and protocol | 24c | Describe and explain any amendments to information provided at registration or in the protocol. | No registration or protocol existed |
| Support | 25 | Describe sources of financial or non-financial support for the review, and the role of the funders or sponsors in the review. | Competing Interests and Funding—no external funding; sponsor role stated. |
| Competing interests | 26 | Declare any competing interests of review authors. | Competing Interests and Funding—commercial NO-related affiliation and other author declarations stated. |
| Availability of data, code and other materials | 27 | Report which of the following are publicly available and where they can be found: template data collection forms; data extracted from included studies; data used for all analyses; analytic code; any other materials used in the review. | Data and Materials Availability statement to accompany the submission; reconstructed S1–S3 supplied with the article. |

# Part B. PRISMA 2020 for Abstracts checklist

| **Section/topic** | **Item** | **PRISMA 2020 item** | **Location in revised report / transparent disposition** |
| --- | --- | --- | --- |
| Title | 1 | Identify the report as a systematic review. | Title |
| Objectives | 2 | Provide an explicit statement of the main objective(s) or question(s) the review addresses. | Abstract—Background |
| Eligibility criteria | 3 | Specify the inclusion and exclusion criteria for the review. | Abstract—Methods |
| Information sources | 4 | Specify the information sources (e.g. databases, registers) used to identify studies and the date when each was last searched. | Abstract—Methods. Databases and coverage are named; exact historical final source-specific dates were not recoverable. |
| Risk of bias | 5 | Specify the methods used to assess risk of bias in the included studies. | Abstract—Methods; Supplementary Table S2 |
| Synthesis of results | 6 | Specify the methods used to present and synthesise results. | Abstract—Methods |
| Included studies | 7 | Give the total number of included studies and participants and summarise relevant characteristics of studies. | Abstract—Methods and Results—54 reports: 21 clinical, 19 animal and 14 in vitro; no aggregate participant total reported. |
| Synthesis of results | 8 | Present results for main outcomes, preferably indicating the number of included studies and participants for each. If meta-analysis was done, report the summary estimate and confidence/credible interval. If comparing groups, indicate the direction of the effect (i.e. which group is favoured). | Abstract—Results |
| Limitations of evidence | 9 | Provide a brief summary of the limitations of the evidence included in the review (e.g. study risk of bias, inconsistency and imprecision). | Abstract—Conclusion/limitations statement |
| Interpretation | 10 | Provide a general interpretation of the results and important implications. | Abstract—Conclusion |
| Funding | 11 | Specify the primary source of funding for the review. | Abstract—Funding—no external funding |
| Registration | 12 | Provide the register name and registration number. | Abstract—Registration—not registered |
