# Supplementary material for: The microbiome–nitric oxide axis in cancer immunotherapy: a targeted qualitative systematic review of mechanistic and translational evidence
Source: Front Immunol. 2026 Sep 24;17:1904698. doi: 10.3389/fimmu.2026.1904698 (PMC13646043; doi:10.3389/fimmu.2026.1904698)
Supplement: Supplementary file 2 [file Table1.docx]

**Supplementary Table S1**

**Characteristics of the clinical, animal and in vitro studies included in the qualitative systematic review**

This table is synchronized with the reconstructed 54-study inventory used in Supplementary Table S2: 21 clinical studies, 19 animal studies and 14 in vitro mechanistic studies. Each publication appears once and is classified according to its principal study design. NR indicates information not reported in the reconstructed source record and requiring confirmation from the full article before final submission.

Evidence-domain legend: D1, microbial taxa and ICI response; D2, immune and metabolic mechanisms; D3, nitric oxide/reactive nitrogen signalling; D4, tumour vascular remodelling and immune infiltration; D5, intervention strategies.

**Table S1A. Characteristics of included clinical studies (n = 21)**

| **ID** | **Study and citation** | **Country/setting** | **Cancer type** | **Design** | **Sample size** | **ICI regimen** | **Microbiome/NO exposure** | **Assessment method** | **Main outcomes** | **Principal finding / synthesis role** | **Domain(s)** |
| --- | --- | --- | --- | --- | --- | --- | --- | --- | --- | --- | --- |
| C01 | Chaput et al., 2017 Chaput N, et al. Baseline gut microbiota predicts clinical response and colitis in metastatic melanoma patients treated with ipilimumab. Ann Oncol. 2017;28:1368–1379. doi:10.1093/annonc/mdx108. | NR | Melanoma | Prospective, single-centre metastatic melanoma cohort; baseline and serial 16S profiling during ipilimumab. | 26 | Ipilimumab / CTLA-4 blockade | Baseline/serial gut microbiome composition | 16S rRNA profiling; Longitudinal sampling | ICI response | Supportive clinical association; interpreted cautiously. | D1; toxicity context |
| C02 | Frankel et al., 2017 Frankel AE, et al. Metagenomic shotgun sequencing and unbiased metabolomic profiling identify specific human gut microbiota and metabolites associated with immune checkpoint therapy efficacy in melanoma patients. Neoplasia. 2017;19:848–855. doi:10.1016/j.neo.2017.08.004. | NR | Melanoma | Prospective exploratory melanoma cohort; shotgun metagenomics and metabolomics across heterogeneous ICI regimens. | NR | ICI regimen heterogeneous/NR | Gut microbiome and microbial metabolites | Shotgun metagenomics; Metabolomics | ICI response | Hypothesis-generating only. | D1; D2 |
| C03 | Routy et al., 2018 Routy B, et al. Gut microbiome influences efficacy of PD-1-based immunotherapy against epithelial tumors. Science. 2018;359:91–97. doi:10.1126/science.aan3706. | NR | Mixed epithelial tumours | Prospective multicancer clinical cohorts assessing antibiotics, stool microbiota and PD-1 outcomes, with mechanistic transfer experiments. | NR | Anti-PD-1 | Antibiotic exposure and gut microbiome | Microbiome profiling method NR | ICI response; PFS/OS; Immune correlates | Primary clinical evidence. | D1; D2; D5 |
| C04 | Gopalakrishnan et al., 2018 Gopalakrishnan V, et al. Gut microbiome modulates response to anti-PD-1 immunotherapy in melanoma patients. Science. 2018;359:97–103. doi:10.1126/science.aan4236. | NR | Melanoma | Prospective metastatic melanoma cohort; baseline 16S/metagenomic profiling linked to anti-PD-1 response and tumour immune features. | NR | Anti-PD-1 | Baseline/serial gut microbiome composition | 16S rRNA profiling; Shotgun metagenomics | ICI response; Immune correlates | Important supportive clinical evidence. | D1; D2 |
| C05 | Matson et al., 2018 Matson V, et al. The commensal microbiome is associated with anti-PD-1 efficacy in metastatic melanoma patients. Science. 2018;359:104–108. doi:10.1126/science.aao3290. | NR | Melanoma | Prospective metastatic melanoma cohort with baseline stool profiling, immunologic correlates and murine FMT validation. | NR | Anti-PD-1 | FMT / donor microbiota | FMT engraftment/immune profiling | ICI response | Important supportive clinical evidence. | D1; D2 |
| C06 | Jin et al., 2019 Jin Y, et al. The diversity of gut microbiome is associated with favorable responses to anti-programmed death 1 immunotherapy in Chinese patients with NSCLC. J Thorac Oncol. 2019;14:1378–1389. doi:10.1016/j.jtho.2019.04.007. | China | NSCLC | Prospective Chinese NSCLC cohort with serial stool sampling during anti-PD-1 treatment. | NR | Anti-PD-1 | Baseline/serial gut microbiome composition | Longitudinal sampling | ICI response | Supportive population-specific evidence. | D1 |
| C07 | Wind et al., 2020 Wind TT, et al. Gut microbial species and metabolic pathways associated with response to treatment with immune checkpoint inhibitors in metastatic melanoma. Melanoma Res. 2020;30:235–246. | NR | Melanoma | Prospective metastatic melanoma cohort using shotgun metagenomics with adjustment for selected microbiome confounders. | NR | ICI regimen heterogeneous/NR | Baseline/serial gut microbiome composition | Shotgun metagenomics | ICI response | Supportive but cohort-specific evidence. | D1; D2 |
| C08 | Song et al., 2020 Song P, et al. Relationship between intestinal flora structure and metabolite analysis and immunotherapy efficacy in Chinese NSCLC patients. Thorac Cancer. 2020;11:1621–1632. doi:10.1111/1759-7714.13442. | China | NSCLC | Prospective/observational Chinese NSCLC cohort examining gut microbiota, metabolites and ICI efficacy. | NR | ICI regimen heterogeneous/NR | Gut microbiome and microbial metabolites | Metabolomics | ICI response | Supportive exploratory evidence. | D1; D2 |
| C09 | Hakozaki et al., 2020 Hakozaki T, et al. The gut microbiome associates with immune checkpoint inhibition outcomes in patients with advanced non-small cell lung cancer. Cancer Immunol Res. 2020;8:1243–1250. doi:10.1158/2326-6066.CIR-20-0196. | Multicentre; country NR | NSCLC | Multicentre advanced NSCLC cohort linking baseline gut microbiota to ICI outcomes. | NR | ICI regimen heterogeneous/NR | Baseline/serial gut microbiome composition | Microbiome profiling method NR | ICI response; PFS/OS | Supportive clinical evidence. | D1 |
| C10 | Peng et al., 2020 Peng Z, et al. The gut microbiome is associated with clinical response to anti-PD-1/PD-L1 immunotherapy in gastrointestinal cancer. Cancer Immunol Res. 2020;8:1251–1261. doi:10.1158/2326-6066.CIR-19-1014. | NR | Gastrointestinal cancers | Prospective gastrointestinal cancer cohort (n≈74) receiving anti-PD-1/PD-L1 therapy; longitudinal microbiome analysis. | 74 | Anti-PD-1/PD-L1 | Baseline/serial gut microbiome composition | Longitudinal sampling | ICI response | Supportive cross-cancer clinical evidence. | D1; D2 |
| C11 | Baruch et al., 2021 Baruch EN, et al. Fecal microbiota transplant promotes response in immunotherapy-refractory melanoma patients. Science. 2021;371:602–609. doi:10.1126/science.abb5920. | NR | Melanoma | Open-label, single-arm pilot FMT plus anti-PD-1 rechallenge in immunotherapy-refractory melanoma. | NR | Anti-PD-1 | FMT / donor microbiota | FMT engraftment/immune profiling | ICI response | Proof-of-concept; not definitive efficacy evidence. | D1; D2; D5 |
| C12 | Davar et al., 2021 Davar D, et al. Fecal microbiota transplant overcomes resistance to anti-PD-1 therapy in melanoma patients. Science. 2021;371:595–602. doi:10.1126/science.abf3363. | NR | Melanoma | Single-arm phase I FMT plus pembrolizumab in anti-PD-1-refractory melanoma with serial multi-omic and immune profiling. | NR | Pembrolizumab | FMT / donor microbiota | Longitudinal sampling; Multi-omics; FMT engraftment/immune profiling | ICI response; Immune correlates | Strong mechanistic proof-of-concept; efficacy remains preliminary. | D1; D2; D5 |
| C13 | Spencer et al., 2021 Spencer CN, et al. Dietary fiber and probiotics influence the gut microbiome and melanoma immunotherapy response. Science. 2021;374:1632–1640. doi:10.1126/science.aaz7015. | NR | Melanoma | Prospective observational melanoma cohort evaluating self-reported diet/probiotic exposure, microbiome and ICI outcomes, with mouse experiments. | NR | ICI regimen heterogeneous/NR | Dietary fibre and probiotic exposure | Microbiome profiling method NR | ICI response; PFS/OS | Supportive association; not evidence for a prescribed intervention. | D1; D2; D5 |
| C14 | Mao et al., 2021 Mao J, et al. Gut microbiome is associated with the clinical response to anti-PD-1-based immunotherapy in hepatobiliary cancers. J Immunother Cancer. 2021;9:e003334. doi:10.1136/jitc-2021-003334. | NR | Hepatobiliary cancers | Prospective hepatobiliary cancer cohort with baseline/serial metagenomic and metabolomic profiling during anti-PD-1 therapy. | NR | Anti-PD-1 | Baseline/serial gut microbiome composition | Shotgun metagenomics; Metabolomics; Longitudinal sampling | ICI response | Supportive tumour-specific evidence. | D1; D2 |
| C15 | Derosa et al., 2022 Derosa L, et al. Intestinal Akkermansia muciniphila predicts clinical response to PD-1 blockade in patients with advanced non-small-cell lung cancer. Nat Med. 2022;28:315–324. doi:10.1038/s41591-021-01655-5. | Multicentre; country NR | NSCLC | Large prospective multicentre NSCLC cohort with validation analyses and quantitative A. muciniphila assessment. | NR | Anti-PD-1 | A. muciniphila abundance | Microbiome profiling method NR | ICI response | Primary clinical evidence. | D1 |
| C16 | Lee et al., 2022 Lee KA, et al. Cross-cohort gut microbiome associations with immune checkpoint inhibitor response in advanced melanoma. Nat Med. 2022;28:535–544. doi:10.1038/s41591-022-01695-5. | Multinational | Melanoma | Multi-cohort, cross-country metagenomic analysis with harmonized reanalysis and external validation. | NR | ICI regimen heterogeneous/NR | Baseline/serial gut microbiome composition | Shotgun metagenomics | ICI response | Primary evidence for cohort dependence and limited universal biomarkers. | D1 |
| C17 | McCulloch et al., 2022 McCulloch JA, et al. Intestinal microbiota signatures of clinical response and immune-related adverse events in melanoma patients treated with anti-PD-1. Nat Med. 2022;28:545–556. doi:10.1038/s41591-022-01698-2. | NR | Melanoma | Prospective melanoma cohort integrated with reanalysis of external datasets and immune/toxicity outcomes. | NR | Anti-PD-1 | Baseline/serial gut microbiome composition | Microbiome profiling method NR | ICI response; PFS/OS; Immune-related toxicity; Immune correlates | Primary clinical evidence. | D1; toxicity context |
| C18 | Newsome et al., 2022 Newsome RC, et al. Interaction of bacterial genera associated with therapeutic response to immune checkpoint PD-1 blockade in a United States cohort. Genome Med. 2022;14:35. doi:10.1186/s13073-022-01037-7. | United States | NSCLC | US NSCLC cohort with microbiome network/interactions analysis and clinical response assessment. | NR | Anti-PD-1 | Baseline/serial gut microbiome composition | Microbiome profiling method NR | ICI response | Supportive cohort-specific evidence. | D1; D2 |
| C19 | Lee et al., 2022 (HCC) Lee PC, et al. Gut microbiota and metabolites associate with outcomes of immune checkpoint inhibitor-treated unresectable hepatocellular carcinoma. J Immunother Cancer. 2022;10:e004779. doi:10.1136/jitc-2022-004779. | NR | Hepatocellular carcinoma | Prospective unresectable HCC discovery and validation cohorts with stool microbiome, bile acids and ICI outcomes. | NR | ICI regimen heterogeneous/NR | Gut microbiome and microbial metabolites | Metabolomics | ICI response; PFS/OS | Supportive tumour-specific clinical evidence. | D1; D2 |
| C20 | Peiffer et al., 2022 Peiffer LB, et al. Composition of gastrointestinal microbiota in association with treatment response in individuals with metastatic castration-resistant prostate cancer progressing on enzalutamide and initiating pembrolizumab. Neoplasia. 2022;32:100822. doi:10.1016/j.neo.2022.100822. | NR | Gastrointestinal cancers | Small prospective pembrolizumab cohort in metastatic castration-resistant prostate cancer with gastrointestinal microbiome profiling. | NR | Pembrolizumab | Baseline/serial gut microbiome composition | Microbiome profiling method NR | ICI response | Hypothesis-generating only. | D1 |
| C21 | Kim et al., 2024 Kim Y, et al. Fecal microbiota transplantation improves anti-PD-1 inhibitor efficacy in unresectable or metastatic solid cancers refractory to anti-PD-1 inhibitor. Cell Host Microbe. 2024;32:1380–1393.e9. | NR | Mixed advanced solid tumours | Prospective single-arm FMT plus continued anti-PD-1 in 13 refractory advanced solid-cancer patients with mechanistic profiling. | NR | Anti-PD-1 | FMT / donor microbiota | FMT engraftment/immune profiling | ICI response; Immune correlates | Proof-of-concept only. | D1; D2; D5 |

**Table S1B. Characteristics of included animal studies (n = 19)**

| **ID** | **Study and citation** | **Animal/tumour model** | **Sample size** | **Intervention/exposure** | **ICI or cancer therapy** | **Comparator** | **Main outcomes** | **Principal finding / synthesis role** | **Domain(s)** |
| --- | --- | --- | --- | --- | --- | --- | --- | --- | --- |
| A01 | Ito et al., 2015 Ito H, et al. Inhibition of inducible nitric oxide synthase enhances the anti-tumor effects of cancer immunotherapy using a TLR7 agonist in mice. Cancer Immunol Immunother. 2015;64:429–436. | Mouse; Mouse tumour-immunotherapy model combining iNOS inhibition with a TLR7 agonist. | NR | NO/NOS pathway modulation | Cancer therapy/model-specific comparator | Vehicle/untreated and/or therapy-alone controls; exact groups NR | Tumour growth/control | Supportive context-dependent NO evidence. | D3; D5 |
| A02 | Ridnour et al., 2015 Ridnour LA, et al. NOS inhibition modulates immune polarization and improves radiation-induced tumor growth delay. Cancer Res. 2015;75:2788–2799. | Mouse; Mouse tumour models assessing NOS inhibition, immune polarization and radiation response. | NR | NO/NOS pathway modulation | Radiotherapy | Vehicle/untreated and/or therapy-alone controls; exact groups NR | Tumour growth/control; Immune-cell phenotype/infiltration | Supportive NO-immunology evidence; indirect to checkpoint blockade. | D3; D4 |
| A03 | Sivan et al., 2015 Sivan A, et al. Commensal Bifidobacterium promotes antitumor immunity and facilitates anti-PD-L1 efficacy. Science. 2015;350:1084–1089. doi:10.1126/science.aac4255. | Mouse; Syngeneic melanoma models comparing microbiota states, fecal transfer and Bifidobacterium administration with anti-PD-L1. | NR | Bifidobacterium / microbiota transfer | Anti-PD-L1 | Model-specific comparator groups | Tumour growth/control | Strong causal preclinical support. | D1; D2; D5 |
| A04 | Vétizou et al., 2015 Vétizou M, et al. Anticancer immunotherapy by CTLA-4 blockade relies on the gut microbiota. Science. 2015;350:1079–1084. doi:10.1126/science.aad1329. | Germ-free/antibiotic-treated mouse; Multiple syngeneic tumour models, germ-free/antibiotic perturbation and defined bacterial reconstitution during CTLA-4 blockade. | NR | Microbiota perturbation/reconstitution | CTLA-4 blockade | Vehicle/untreated and/or therapy-alone controls; exact groups NR | Tumour growth/control | Strong preclinical support, tempered by reporting limitations. | D1; D2; D5 |
| A05 | Fauskanger et al., 2018 Fauskanger M, Haabeth OAW, Skjeldal FM, Bogen B, Tveita AA. Tumor killing by CD4+ T cells is mediated via induction of inducible nitric oxide synthase-dependent macrophage cytotoxicity. Front Immunol. 2018;9:1684. doi:10.3389/fimmu.2018.01684. | Mouse; Syngeneic lymphoma and melanoma models examining antigen-specific CD4 T-cell recognition, macrophage iNOS induction and tumour rejection. | NR | NO/NOS pathway modulation | Cancer therapy/model-specific comparator | Vehicle/untreated and/or therapy-alone controls; exact groups NR | Tumour growth/control; Immune-cell phenotype/infiltration | Strong mechanistic animal evidence for macrophage-derived NO in antitumour immunity. | D2; D3; D4 |
| A06 | Sung et al., 2019 Sung YC, et al. Delivery of nitric oxide with a nanocarrier promotes tumour vessel normalization and potentiates anti-cancer therapies. Nat Nanotechnol. 2019;14:1160–1169. | Mouse; Multiple tumour models with localized NO nanocarrier delivery, perfusion/vascular measurements and combination therapy. | NR | NO/NOS pathway modulation | Cancer therapy/model-specific comparator | Vehicle/untreated and/or therapy-alone controls; exact groups NR | Tumour growth/control; Perfusion/vascular endpoints | High-weight preclinical vascular evidence; not microbiota-derived NO. | D3; D4; D5 |
| A07 | Tanoue et al., 2019 Tanoue T, et al. A defined commensal consortium elicits CD8 T cells and anti-cancer immunity. Nature. 2019;565:600–605. doi:10.1038/s41586-019-0878-z. | Gnotobiotic mouse; Gnotobiotic mice colonized with a defined 11-strain consortium; immune phenotyping, infection and tumour challenge models. | NR | Defined commensal consortium | Cancer therapy/model-specific comparator | Vehicle/untreated and/or therapy-alone controls; exact groups NR | Tumour growth/control; Immune-cell phenotype/infiltration | High-weight preclinical mechanistic evidence. | D2; D5 |
| A08 | Coutzac et al., 2020 Coutzac C, et al. Systemic short-chain fatty acids limit antitumor effect of CTLA-4 blockade in hosts with cancer. Nat Commun. 2020;11:2168. doi:10.1038/s41467-020-16079-x. | Mouse; Mouse tumour models testing systemic SCFAs during CTLA-4 blockade, integrated with patient biomarker observations. | NR | SCFA/butyrate exposure | CTLA-4 blockade | Vehicle/untreated and/or therapy-alone controls; exact groups NR | Tumour growth/control | Important contradictory/context-dependent preclinical evidence. | D2; D5 |
| A09 | Shi et al., 2020 Shi L, et al. Combining IL-2-based immunotherapy with commensal probiotics produces enhanced antitumor immune response and tumor clearance. J Immunother Cancer. 2020;8:e000973. | Mouse; Syngeneic tumour models combining commensal probiotics with IL-2-based immunotherapy. | NR | Commensal probiotic intervention | IL-2-based immunotherapy | Vehicle/untreated and/or therapy-alone controls; exact groups NR | Tumour growth/control | Supportive intervention evidence; indirect to ICI. | D2; D5 |
| A10 | Xu et al., 2020 Xu J, Luo Y, Yuan C, Han L, Wu Q, Xu L, et al. Downregulation of nitric oxide collaborated with radiotherapy to promote anti-tumor immune response via inducing CD8+ T-cell infiltration. Int J Biol Sci. 2020;16:1563–1574. doi:10.7150/ijbs.41653. | Mouse; Murine tumour models combining NO pathway suppression with radiotherapy and assessment of CD8 T-cell infiltration and tumour control. | NR | NO/NOS pathway modulation | Radiotherapy | Vehicle/untreated and/or therapy-alone controls; exact groups NR | Tumour growth/control; Immune-cell phenotype/infiltration | Supportive context-dependent NO and immune-access evidence; indirect to ICI. | D3; D4; D5 |
| A11 | Fan et al., 2021 Fan L, et al. A. muciniphila suppresses colorectal tumorigenesis by inducing TLR2/NLRP3-mediated M1-like tumour-associated macrophages. Cancer Immunol Res. 2021;9:1111–1124. | Mouse; Colorectal tumour models with A. muciniphila administration, macrophage phenotyping and pathway perturbation. | NR | Microbiome/metabolite intervention | Cancer therapy/model-specific comparator | Vehicle/untreated and/or therapy-alone controls; exact groups NR | Tumour growth/control; Immune-cell phenotype/infiltration | Supportive mechanistic animal evidence. | D1; D2; D3 |
| A12 | Luu et al., 2021 Luu M, et al. Microbial short-chain fatty acids modulate CD8+ T cell responses and improve adoptive immunotherapy for cancer. Nat Commun. 2021;12:4077. | Mouse; Mouse tumour/adoptive immunotherapy models with controlled SCFA exposure and CD8 T-cell analyses. | NR | SCFA/butyrate exposure | Adoptive T-cell therapy | Vehicle/untreated and/or therapy-alone controls; exact groups NR | Tumour growth/control; Immune-cell phenotype/infiltration | Strong supportive metabolite–T-cell evidence; indirect to ICI. | D2; D5 |
| A13 | Hezaveh et al., 2022 Hezaveh K, et al. Tryptophan-derived microbial metabolites activate the aryl hydrocarbon receptor in tumour-associated macrophages to suppress anti-tumour immunity. Immunity. 2022;55:324–340.e8. | Mouse; Multiple tumour models with microbial-metabolite manipulation, AhR perturbation and macrophage-specific validation. | NR | Tryptophan-derived microbial metabolites | Cancer therapy/model-specific comparator | Vehicle/untreated and/or therapy-alone controls; exact groups NR | Tumour growth/control; Immune-cell phenotype/infiltration; Metabolic endpoints | High-value mechanistic evidence, with standard animal-reporting limitations. | D2 |
| A14 | Confino et al., 2023 Confino H, et al. Intratumoral administration of high-concentration nitric oxide and anti-mPD-1 treatment improves tumor regression rates and survival in CT26 tumour-bearing mice. Cells. 2023;12:2439. doi:10.3390/cells12202439. | BALB/c mouse; BALB/c bilateral CT26 model testing intratumoral gaseous NO (50,000 ppm) plus systemic anti-mPD-1. | NR | NO/NOS pathway modulation | Anti-mPD-1 | Vehicle/untreated and/or therapy-alone controls; exact groups NR | Tumour growth/control | Preliminary proof-of-concept only. | D3; D5 |
| A15 | Cunha et al., 2023 Cunha PP, Bargiela D, Minogue E, Krause LCM, Barbieri L, Brombach C, et al. Infiltration of tumors is regulated by T cell-intrinsic nitric oxide synthesis. Cancer Immunol Res. 2023;11:351–363. doi:10.1158/2326-6066.CIR-22-0387. | Mouse; T-cell-specific Nos2 deletion models, adoptive transfer and tumour-infiltration experiments examining endogenous T-cell NO in antitumour immunity. | NR | NO/NOS pathway modulation | Adoptive T-cell therapy | Vehicle/untreated and/or therapy-alone controls; exact groups NR | Tumour growth/control; Immune-cell phenotype/infiltration | High-value mechanistic animal evidence, interpreted with standard reporting limitations. | D2; D3; D4 |
| A16 | Zhu et al., 2023 Zhu X, et al. Microbial metabolite butyrate promotes anti-PD-1 antitumor efficacy by modulating T-cell receptor signaling of cytotoxic CD8 T cells. Gut Microbes. 2023;15:2249143. | Mouse; Mouse tumour models combining butyrate and anti-PD-1, with CD8 T-cell functional and epigenetic analyses. | NR | SCFA/butyrate exposure | Anti-PD-1 | Vehicle/untreated and/or therapy-alone controls; exact groups NR | Tumour growth/control; Immune-cell phenotype/infiltration | Direct supportive preclinical ICI evidence. | D2; D5 |
| A17 | Fang et al., 2024 Fang J, et al. Akkermansia muciniphila improves gastric cancer treatment by modulating the immune microenvironment. Future Microbiol. 2024;19:481–494. | Mouse; Gastric cancer model testing A. muciniphila and immune-microenvironment changes. | NR | A. muciniphila administration | Cancer therapy/model-specific comparator | Vehicle/untreated and/or therapy-alone controls; exact groups NR | Tumour growth/control; Immune-cell phenotype/infiltration | Hypothesis-generating preclinical evidence. | D1; D2 |
| A18 | Lan et al., 2024 Lan X, et al. Akkermansia muciniphila might improve anti-PD-1 therapy against HCC by changing host bile acid metabolism. J Gene Med. 2024;26:e3639. | Mouse; Hepatocellular carcinoma mouse model testing A. muciniphila, bile-acid metabolism and anti-PD-1 response. | NR | A. muciniphila administration | Anti-PD-1 | Vehicle/untreated and/or therapy-alone controls; exact groups NR | Tumour growth/control; Metabolic endpoints | Supportive tumour-specific preclinical evidence. | D1; D2; D5 |
| A19 | Zhu et al., 2024 Zhu Z, et al. Landscape of tumoral ecosystem for enhanced anti-PD-1 immunotherapy by gut Akkermansia muciniphila. Cell Rep. 2024;43. | Mouse; Integrated mouse tumour, microbiome and tumour-ecosystem analyses testing A. muciniphila with anti-PD-1. | NR | A. muciniphila administration | Anti-PD-1 | Vehicle/untreated and/or therapy-alone controls; exact groups NR | Tumour growth/control | Strong supportive preclinical evidence. | D1; D2; D4; D5 |

**Table S1C. Characteristics of included in vitro mechanistic studies (n = 14)**

| **ID** | **Study and citation** | **Cell/tissue model** | **Exposure** | **Concentration/duration** | **Comparator/control** | **Mechanistic endpoint** | **Principal finding / synthesis role** | **Domain(s)** |
| --- | --- | --- | --- | --- | --- | --- | --- | --- |
| V01 | Segain et al., 2000 Segain JP, et al. Butyrate inhibits inflammatory responses through NF-κB inhibition: implications for Crohn's disease. Gut. 2000;47:397–403. | Intestinal tissue/cellular inflammatory systems treated with butyrate; NF-κB and cytokine endpoints. | Butyrate | NR in reconstructed source record | Vehicle/biological controls; details NR | NF-κB signalling | Contextual mechanistic support. | D2 (indirect) |
| V02 | Menzel et al., 2004 Menzel T, et al. Butyrate inhibits leukocyte adhesion to endothelial cells via modulation of VCAM-1. Inflamm Bowel Dis. 2004;10:122–128. | Endothelial–leukocyte adhesion assays with butyrate exposure. | Butyrate | NR in reconstructed source record | Vehicle/biological controls; details NR | Endothelial/leukocyte adhesion | Illustrative only. | D4 (indirect) |
| V03 | Zapolska-Downar et al., 2004 Zapolska-Downar D, et al. Butyrate inhibits cytokine-induced VCAM-1 and ICAM-1 expression in cultured endothelial cells: the role of NF-κB and PPARα. J Nutr Biochem. 2004;15:220–228. | Cytokine-stimulated endothelial cells treated with butyrate; adhesion molecules and NF-κB/PPARα assays. | Butyrate | NR in reconstructed source record | Stimulated cells with vehicle/untreated controls | NF-κB signalling; Endothelial/leukocyte adhesion | Mechanistic plausibility only. | D4 (indirect) |
| V04 | Miller et al., 2005 Miller SJ, et al. Short-chain fatty acids modulate gene expression for vascular endothelial cell adhesion molecules. Nutrition. 2005;21:740–748. | Cultured vascular endothelial cells exposed to SCFAs; adhesion-molecule expression endpoints. | Short-chain fatty acids | NR in reconstructed source record | Vehicle/biological controls; details NR | Endothelial/leukocyte adhesion | Mechanistic plausibility only. | D4 (indirect) |
| V05 | Nagaraj et al., 2007 Nagaraj S, Gupta K, Pisarev V, Kinarsky L, Sherman S, Kang L, et al. Altered recognition of antigen is a mechanism of CD8+ T-cell tolerance in cancer. Nat Med. 2007;13:828–835. doi:10.1038/nm1609. | MDSC–T-cell coculture, peptide–MHC binding and biochemical nitration analyses examining peroxynitrite-mediated modification of the TCR–CD8 complex. | Nitrite | NR in reconstructed source record | Matched coculture and pathway-inhibition controls | T-cell function/signalling; Tumour-cell response | High-value mechanistic evidence for reactive nitrogen species-mediated T-cell dysfunction. | D2; D3 |
| V06 | Molon et al., 2011 Molon B, Ugel S, Del Pozzo F, Soldani C, Zilio S, Avella D, et al. Chemokine nitration prevents intratumoral infiltration of antigen-specific T cells. J Exp Med. 2011;208:1949–1962. doi:10.1084/jem.20101956. | Biochemical chemokine-nitration assays, T-cell chemotaxis systems and human/murine tumour material examining reactive nitrogen species-mediated exclusion of tumour-specific T cells. | Reactive nitrogen species/peroxynitrite | NR in reconstructed source record | Vehicle/biological controls; details NR | T-cell function/signalling; Chemotaxis/immune-cell trafficking; Tumour-cell response | High-value mechanistic evidence linking reactive nitrogen species to immune exclusion. | D3; D4 |
| V07 | Mundy-Bosse et al., 2011 Mundy-Bosse BL, Lesinski GB, Jaime-Ramirez AC, Benninger K, Khan M, Kuppusamy P, et al. Myeloid-derived suppressor cell inhibition of the IFN response in tumour-bearing mice. Cancer Res. 2011;71:5101–5110. doi:10.1158/0008-5472.CAN-10-2670. | Ex vivo and in vitro MDSC–immune-cell systems assessing NO-dependent impairment of IFN signalling, including Nos2-deficient and pharmacologic controls. | NO/iNOS pathway manipulation | NR in reconstructed source record | Vehicle/biological controls; details NR | Myeloid/macrophage phenotype; Tumour-cell response | High-value mechanistic evidence for NO-mediated impairment of interferon responsiveness. | D2; D3 |
| V08 | Jiang et al., 2012 Jiang H, et al. Concentration- and stage-specific effects of nitrite on colon cancer cell lines. Nitric Oxide. 2012;26:267–273. | Colon cancer cell lines exposed to graded nitrite concentrations across disease-stage phenotypes; proliferation/invasion-related outcomes. | Nitrite | NR in reconstructed source record | Dose-gradient and untreated controls | Tumour-cell response | Supports biphasic/context-dependent nitrite biology only. | D3 |
| V09 | Raber et al., 2014 Raber PL, Thevenot P, Sierra R, Wyczechowska D, Halle D, Ramirez ME, et al. Subpopulations of myeloid-derived suppressor cells impair T-cell responses through independent nitric oxide-related pathways. Int J Cancer. 2014;134:2853–2864. doi:10.1002/ijc.28622. | Tumour-derived granulocytic and monocytic MDSC cocultures with activated T cells, using NO scavenging, iNOS knockdown and peroxynitrite pathway interrogation. | Nitrite | NR in reconstructed source record | Matched coculture and pathway-inhibition controls | T-cell function/signalling; Myeloid/macrophage phenotype; Tumour-cell response | High-value mechanistic evidence for distinct NO-related immunosuppressive pathways. | D2; D3 |
| V10 | Lu et al., 2015 Lu G, et al. Myeloid cell-derived inducible nitric oxide synthase suppresses M1 macrophage polarization. Nat Commun. 2015;6:6676. | Primary/derived myeloid cells; iNOS manipulation, IRF5 nitration and macrophage-polarization assays. | Reactive nitrogen species/peroxynitrite | NR in reconstructed source record | Vehicle/biological controls; details NR | Myeloid/macrophage phenotype | High-value mechanistic evidence. | D2; D3 |
| V11 | Bolduc et al., 2017 Bolduc JF, et al. Epigenetic metabolite acetate inhibits class I/II histone deacetylases, promotes histone acetylation, and increases HIV-1 integration in CD4+ T cells. J Virol. 2017;91:e00524-17. | Cultured CD4 T cells exposed to acetate; HDAC inhibition, histone acetylation and transcriptional outcomes. | Acetate | NR in reconstructed source record | Vehicle/biological controls; details NR | T-cell function/signalling; Epigenetic/HDAC endpoints | Contextual mechanistic support only. | D2 (indirect) |
| V12 | Li et al., 2018 Li M, et al. The anti-inflammatory effects of short-chain fatty acids on LPS- or TNF-α-stimulated endothelial cells via activation of GPR41/43 and inhibition of HDACs. Front Pharmacol. 2018;9:533. | Stimulated endothelial-cell systems exposed to SCFAs with receptor and HDAC pathway analyses. | Short-chain fatty acids | NR in reconstructed source record | Stimulated cells with vehicle/untreated controls | Epigenetic/HDAC endpoints | Supportive mechanistic evidence. | D2; D4 (indirect) |
| V13 | Stiff et al., 2018 Stiff A, Trikha P, Mundy-Bosse B, McMichael E, Mace TA, Benner B, et al. Nitric oxide production by myeloid-derived suppressor cells plays a role in impairing Fc receptor-mediated natural killer cell function. Clin Cancer Res. 2018;24:1891–1904. doi:10.1158/1078-0432.CCR-17-0691. | Human and murine MDSC–NK-cell cocultures assessing NO-dependent impairment of Fc receptor signalling and antibody-dependent cellular cytotoxicity. | NO/iNOS pathway manipulation | NR in reconstructed source record | Matched coculture and pathway-inhibition controls | T-cell function/signalling; NK-cell cytotoxicity; Myeloid/macrophage phenotype | High-value mechanistic evidence for NO-mediated suppression of innate antitumour effector function. | D2; D3 |
| V14 | Wang et al., 2020 Wang Y, et al. Butyrate mitigates TNF-α-induced attachment of monocytes to endothelial cells. J Bioenerg Biomembr. 2020;52:247–256. | TNF-α-stimulated endothelial cells and monocyte-adhesion assays with butyrate. | Butyrate | NR in reconstructed source record | Stimulated cells with vehicle/untreated controls | Endothelial/leukocyte adhesion | Supportive mechanistic evidence. | D4 (indirect) |

**Abbreviations and reporting notes**

FMT, fecal microbiota transplantation; HCC, hepatocellular carcinoma; ICI, immune checkpoint inhibitor; mCRPC, metastatic castration-resistant prostate cancer; NSCLC, non-small-cell lung cancer; NO, nitric oxide; NOS/iNOS, nitric oxide synthase/inducible nitric oxide synthase; PD-1/PD-L1, programmed cell death protein 1/programmed death-ligand 1; SCFA, short-chain fatty acid.

Sample sizes, intervention concentrations and comparator details recorded as NR should be verified against the full article and supplementary methods before journal submission.

The table describes study characteristics and evidentiary contribution; methodological-quality and risk-of-bias judgments are reported separately in Supplementary Table S2.
