# Supplementary material for: The microbiome–nitric oxide axis in cancer immunotherapy: a targeted qualitative systematic review of mechanistic and translational evidence
Source: Front Immunol. 2026 Sep 24;17:1904698. doi: 10.3389/fimmu.2026.1904698 (PMC13646043; doi:10.3389/fimmu.2026.1904698)
Supplement: Supplementary file 3 [file Table2.docx]

**Supplementary Table S2**

**Retrospectively reconstructed study-level methodological appraisal**

**Reconstruction provenance.** The original reviewer-specific screening and appraisal forms were unavailable during revision. The 54-study inventory was therefore reconstructed from the manuscript citation corpus, cited primary reports and accessible supporting material and cross-checked against the revised reference list and Supplementary Table S1. The resulting inventory contains 54 distinct peer-reviewed primary reports: 21 clinical, 19 animal and 14 in vitro mechanistic studies, with each publication represented once according to its principal design**.**

**Participant accounting.** An aggregate clinical participant total is not reported because several cross-cohort analyses, validation datasets and secondary reanalyses may include overlapping participants. Cancer types are reported at study level rather than summed across mixed or overlapping cohorts.

**Appraisal provenance.** The original reviewer-specific appraisal forms were unavailable during revision. Accordingly, two reviewers independently repeated the study-level methodological appraisal using the reconstructed 54-study inventory and accessible full reports and supplementary materials. Domain-level judgments were recorded independently and disagreements were resolved by consensus, with third-reviewer adjudication where required. The resulting revision-stage appraisal forms and consensus decisions were retained in the review archive. These assessments constitute a post hoc reappraisal and are not presented as recovered original reviewer forms.

**Appraisal framework and coding**

Clinical observational studies were assessed using Newcastle–Ottawa Scale (NOS) domains. Scores of 7–9 were classified as high methodological quality, 5–6 as moderate quality and 0–4 as low quality. Non-randomized microbiota interventions were assessed using ROBINS-I. Animal studies were assessed using the SYRCLE domains: sequence generation (SG), baseline characteristics (BC), allocation concealment (AC), random housing (RH), blinding of caregivers/personnel (BP), random outcome assessment (RO), blinded outcome assessment (BO), incomplete outcome data (ID), selective reporting (SR) and other bias (OB). L, U and H denote low, unclear and high risk. Because incomplete reporting was common, a low overall animal-study judgment required all key domains to be low risk; otherwise the overall judgment was unclear unless at least one material domain was high risk.

In vitro mechanistic studies were assessed using a structured eight-domain rubric: appropriate controls (C), biological replication (R), model authentication/characterization (M), exposure characterization (E), objective or blinded/automated outcome assessment where feasible (O), complete statistical reporting (S), low likelihood of selective reporting (P) and orthogonal or rescue validation (V). Y, P and U denote met, partially met and unclear/not reported. Studies meeting 7–8 criteria were categorized as meeting most criteria, those meeting 4–6 as having some limitations, and those meeting three or fewer—or having a critical deficiency—were categorized as having important limitations.

**Evidence-domain legend:** D1, microbial taxa and ICI response; D2, immune and metabolic mechanisms; D3, nitric oxide and reactive nitrogen signalling; D4, tumour vascular remodelling and immune infiltration; D5, intervention strategies.

**Aggregate appraisal summary**

| **Evidence stratum** | **Favourable / low risk** | **Intermediate / unclear** | **Unfavourable / high risk** | **Total** |
| --- | --- | --- | --- | --- |
| Clinical | 5 | 11 | 2 low quality; 3 serious ROBINS-I | 21 |
| Animal | 0 | 17 | 2 | 19 |
| In vitro | 6 | 7 | 1 | 14 |

Appraisal summary. The reconstructed clinical appraisal classified 5 studies as high methodological quality, 11 as moderate quality, 2 as low quality and 3 non-randomized intervention studies as having serious risk of bias. Among animal studies, none met low overall risk across all SYRCLE domains, 17 were judged unclear and 2 high risk. Among in vitro studies, 6 met most criteria, 7 had some limitations and 1 had important limitations. These categories are descriptive and were used to calibrate narrative emphasis rather than to calculate a pooled effect.

**Table S2A. Clinical study-level judgments (21 distinct reports)**

Observational reports were evaluated using the NOS; early single-arm FMT studies were evaluated using ROBINS-I. Cross-cohort and external validation analyses were retained as distinct publications but were not summed to produce an aggregate participant count.

| **Study and full citation** | **Design and eligibility basis** | **Domain-level appraisal** | **Overall judgment** | **Principal methodological considerations** | **Domain(s)** | **Weight in synthesis** |
| --- | --- | --- | --- | --- | --- | --- |
| C01 Chaput et al., 2017 Chaput N, et al. Baseline gut microbiota predicts clinical response and colitis in metastatic melanoma patients treated with ipilimumab. Ann Oncol. 2017;28:1368–1379. doi:10.1093/annonc/mdx108. | Prospective, single-centre metastatic melanoma cohort; baseline and serial 16S profiling during ipilimumab. | NOS: Selection 3/4; Comparability 1/2; Outcome 2/3; total 6/9. | Moderate methodological quality | Small cohort (n=26), exploratory clustering and multiple microbial comparisons; limited control of diet, antibiotics and other microbiome confounders; no external validation. | D1; toxicity context | Supportive clinical association; interpreted cautiously. |
| C02 Frankel et al., 2017 Frankel AE, et al. Metagenomic shotgun sequencing and unbiased metabolomic profiling identify specific human gut microbiota and metabolites associated with immune checkpoint therapy efficacy in melanoma patients. Neoplasia. 2017;19:848–855. doi:10.1016/j.neo.2017.08.004. | Prospective exploratory melanoma cohort; shotgun metagenomics and metabolomics across heterogeneous ICI regimens. | NOS: Selection 3/4; Comparability 0/2; Outcome 1/3; total 4/9. | Low methodological quality | Small, clinically heterogeneous sample; treatment regimens and response groups were mixed; limited multivariable adjustment and no independent validation; high multiplicity relative to sample size. | D1; D2 | Hypothesis-generating only. |
| C03 Routy et al., 2018 Routy B, et al. Gut microbiome influences efficacy of PD-1-based immunotherapy against epithelial tumors. Science. 2018;359:91–97. doi:10.1126/science.aan3706. | Prospective multicancer clinical cohorts assessing antibiotics, stool microbiota and PD-1 outcomes, with mechanistic transfer experiments. | NOS: Selection 4/4; Comparability 2/2; Outcome 2/3; total 8/9. | High methodological quality | Non-randomized exposure to antibiotics and potential residual confounding; heterogeneous tumour types and treatments. Strengths include clinically defined outcomes, multivariable analyses and mechanistic validation. | D1; D2; D5 | Primary clinical evidence. |
| C04 Gopalakrishnan et al., 2018 Gopalakrishnan V, et al. Gut microbiome modulates response to anti-PD-1 immunotherapy in melanoma patients. Science. 2018;359:97–103. doi:10.1126/science.aan4236. | Prospective metastatic melanoma cohort; baseline 16S/metagenomic profiling linked to anti-PD-1 response and tumour immune features. | NOS: Selection 3/4; Comparability 1/2; Outcome 2/3; total 6/9. | Moderate methodological quality | Modest cohort, multiple taxonomic comparisons and limited adjustment for diet/medications; responder definition and sequencing analyses were prespecified, with independent biological validation. | D1; D2 | Important supportive clinical evidence. |
| C05 Matson et al., 2018 Matson V, et al. The commensal microbiome is associated with anti-PD-1 efficacy in metastatic melanoma patients. Science. 2018;359:104–108. doi:10.1126/science.aao3290. | Prospective metastatic melanoma cohort with baseline stool profiling, immunologic correlates and murine FMT validation. | NOS: Selection 3/4; Comparability 1/2; Outcome 2/3; total 6/9. | Moderate methodological quality | Small single-centre cohort and risk of overfitting taxa to response; limited adjustment for microbiome confounders; biological transfer experiments strengthen plausibility. | D1; D2 | Important supportive clinical evidence. |
| C06 Jin et al., 2019 Jin Y, et al. The diversity of gut microbiome is associated with favorable responses to anti-programmed death 1 immunotherapy in Chinese patients with NSCLC. J Thorac Oncol. 2019;14:1378–1389. doi:10.1016/j.jtho.2019.04.007. | Prospective Chinese NSCLC cohort with serial stool sampling during anti-PD-1 treatment. | NOS: Selection 3/4; Comparability 1/2; Outcome 2/3; total 6/9. | Moderate methodological quality | Small cohort and single geographic setting; limited adjustment for diet, antibiotics and tumour molecular covariates; repeated sampling and clinically relevant outcomes are strengths. | D1 | Supportive population-specific evidence. |
| C07 Wind et al., 2020 Wind TT, et al. Gut microbial species and metabolic pathways associated with response to treatment with immune checkpoint inhibitors in metastatic melanoma. Melanoma Res. 2020;30:235–246. | Prospective metastatic melanoma cohort using shotgun metagenomics with adjustment for selected microbiome confounders. | NOS: Selection 3/4; Comparability 1/2; Outcome 1/3; total 5/9. | Moderate methodological quality | Small cohort and exploratory high-dimensional analyses; limited power for multivariable modelling and no external validation. | D1; D2 | Supportive but cohort-specific evidence. |
| C08 Song et al., 2020 Song P, et al. Relationship between intestinal flora structure and metabolite analysis and immunotherapy efficacy in Chinese NSCLC patients. Thorac Cancer. 2020;11:1621–1632. doi:10.1111/1759-7714.13442. | Prospective/observational Chinese NSCLC cohort examining gut microbiota, metabolites and ICI efficacy. | NOS: Selection 3/4; Comparability 1/2; Outcome 1/3; total 5/9. | Moderate methodological quality | Single-centre, modest sample and exploratory analyses; incomplete control of dietary, antibiotic and clinical confounding; external reproducibility uncertain. | D1; D2 | Supportive exploratory evidence. |
| C09 Hakozaki et al., 2020 Hakozaki T, et al. The gut microbiome associates with immune checkpoint inhibition outcomes in patients with advanced non-small cell lung cancer. Cancer Immunol Res. 2020;8:1243–1250. doi:10.1158/2326-6066.CIR-20-0196. | Multicentre advanced NSCLC cohort linking baseline gut microbiota to ICI outcomes. | NOS: Selection 3/4; Comparability 1/2; Outcome 2/3; total 6/9. | Moderate methodological quality | Observational design, modest cohort and potential confounding by antibiotics, proton-pump inhibitors, diet and performance status; clinically standardized outcomes support validity. | D1 | Supportive clinical evidence. |
| C10 Peng et al., 2020 Peng Z, et al. The gut microbiome is associated with clinical response to anti-PD-1/PD-L1 immunotherapy in gastrointestinal cancer. Cancer Immunol Res. 2020;8:1251–1261. doi:10.1158/2326-6066.CIR-19-1014. | Prospective gastrointestinal cancer cohort (n≈74) receiving anti-PD-1/PD-L1 therapy; longitudinal microbiome analysis. | NOS: Selection 3/4; Comparability 1/2; Outcome 2/3; total 6/9. | Moderate methodological quality | Tumour-site heterogeneity, non-randomized design and multiple taxonomic comparisons; residual clinical and dietary confounding; longitudinal sampling is a strength. | D1; D2 | Supportive cross-cancer clinical evidence. |
| C11 Baruch et al., 2021 Baruch EN, et al. Fecal microbiota transplant promotes response in immunotherapy-refractory melanoma patients. Science. 2021;371:602–609. doi:10.1126/science.abb5920. | Open-label, single-arm pilot FMT plus anti-PD-1 rechallenge in immunotherapy-refractory melanoma. | ROBINS-I: confounding Serious; selection Serious; intervention classification Low; deviations Moderate; missing data Moderate; outcome measurement Moderate; reporting Moderate. | Serious risk of bias | Very small, uncontrolled intervention; donor and recipient selection, regression to the mean and continued anti-PD-1 confound causal attribution; no blinding. | D1; D2; D5 | Proof-of-concept; not definitive efficacy evidence. |
| C12 Davar et al., 2021 Davar D, et al. Fecal microbiota transplant overcomes resistance to anti-PD-1 therapy in melanoma patients. Science. 2021;371:595–602. doi:10.1126/science.abf3363. | Single-arm phase I FMT plus pembrolizumab in anti-PD-1-refractory melanoma with serial multi-omic and immune profiling. | ROBINS-I: confounding Serious; selection Serious; intervention classification Low; deviations Moderate; missing data Moderate; outcome measurement Moderate; reporting Moderate. | Serious risk of bias | Small uncontrolled study, selected donors/recipients and no counterfactual comparator; mechanistic correlates are extensive but cannot eliminate confounding or selection bias. | D1; D2; D5 | Strong mechanistic proof-of-concept; efficacy remains preliminary. |
| C13 Spencer et al., 2021 Spencer CN, et al. Dietary fiber and probiotics influence the gut microbiome and melanoma immunotherapy response. Science. 2021;374:1632–1640. doi:10.1126/science.aaz7015. | Prospective observational melanoma cohort evaluating self-reported diet/probiotic exposure, microbiome and ICI outcomes, with mouse experiments. | NOS: Selection 3/4; Comparability 1/2; Outcome 2/3; total 6/9. | Moderate methodological quality | Diet and probiotic use were not randomized and were self-reported; residual healthy-user and socioeconomic confounding; mechanistic mouse experiments support plausibility. | D1; D2; D5 | Supportive association; not evidence for a prescribed intervention. |
| C14 Mao et al., 2021 Mao J, et al. Gut microbiome is associated with the clinical response to anti-PD-1-based immunotherapy in hepatobiliary cancers. J Immunother Cancer. 2021;9:e003334. doi:10.1136/jitc-2021-003334. | Prospective hepatobiliary cancer cohort with baseline/serial metagenomic and metabolomic profiling during anti-PD-1 therapy. | NOS: Selection 3/4; Comparability 1/2; Outcome 2/3; total 6/9. | Moderate methodological quality | Modest sample, heterogeneous hepatobiliary diagnoses and therapies, multiple omic comparisons and limited external validation. | D1; D2 | Supportive tumour-specific evidence. |
| C15 Derosa et al., 2022 Derosa L, et al. Intestinal Akkermansia muciniphila predicts clinical response to PD-1 blockade in patients with advanced non-small-cell lung cancer. Nat Med. 2022;28:315–324. doi:10.1038/s41591-021-01655-5. | Large prospective multicentre NSCLC cohort with validation analyses and quantitative A. muciniphila assessment. | NOS: Selection 4/4; Comparability 2/2; Outcome 2/3; total 8/9. | High methodological quality | Observational exposure and residual confounding remain; abundance-response relation was non-linear and may be population specific. Large sample, standardized outcomes and validation strengthen inference. | D1 | Primary clinical evidence. |
| C16 Lee et al., 2022 Lee KA, et al. Cross-cohort gut microbiome associations with immune checkpoint inhibitor response in advanced melanoma. Nat Med. 2022;28:535–544. doi:10.1038/s41591-022-01695-5. | Multi-cohort, cross-country metagenomic analysis with harmonized reanalysis and external validation. | NOS: Selection 4/4; Comparability 2/2; Outcome 2/3; total 8/9. | High methodological quality | Between-cohort heterogeneity, batch effects and residual diet/medication confounding; cross-cohort design and validation substantially reduce single-cohort bias. | D1 | Primary evidence for cohort dependence and limited universal biomarkers. |
| C17 McCulloch et al., 2022 McCulloch JA, et al. Intestinal microbiota signatures of clinical response and immune-related adverse events in melanoma patients treated with anti-PD-1. Nat Med. 2022;28:545–556. doi:10.1038/s41591-022-01698-2. | Prospective melanoma cohort integrated with reanalysis of external datasets and immune/toxicity outcomes. | NOS: Selection 4/4; Comparability 2/2; Outcome 2/3; total 8/9. | High methodological quality | Observational design and heterogeneity across external datasets; taxonomic signals remain context dependent. Multi-cohort validation and integrated analyses strengthen reliability. | D1; toxicity context | Primary clinical evidence. |
| C18 Newsome et al., 2022 Newsome RC, et al. Interaction of bacterial genera associated with therapeutic response to immune checkpoint PD-1 blockade in a United States cohort. Genome Med. 2022;14:35. doi:10.1186/s13073-022-01037-7. | US NSCLC cohort with microbiome network/interactions analysis and clinical response assessment. | NOS: Selection 3/4; Comparability 1/2; Outcome 2/3; total 6/9. | Moderate methodological quality | Modest cohort and high-dimensional interaction modelling; limited external validation and potential residual confounding; analytic transparency and functional interpretation are strengths. | D1; D2 | Supportive cohort-specific evidence. |
| C19 Lee et al., 2022 (HCC) Lee PC, et al. Gut microbiota and metabolites associate with outcomes of immune checkpoint inhibitor-treated unresectable hepatocellular carcinoma. J Immunother Cancer. 2022;10:e004779. doi:10.1136/jitc-2022-004779. | Prospective unresectable HCC discovery and validation cohorts with stool microbiome, bile acids and ICI outcomes. | NOS: Selection 4/4; Comparability 2/2; Outcome 2/3; total 8/9. | High methodological quality | Non-randomized design, modest validation sample and multiple microbiome/metabolite comparisons; liver disease severity and concurrent treatments may confound outcomes. | D1; D2 | Supportive tumour-specific clinical evidence. |
| C20 Peiffer et al., 2022 Peiffer LB, et al. Composition of gastrointestinal microbiota in association with treatment response in individuals with metastatic castration-resistant prostate cancer progressing on enzalutamide and initiating pembrolizumab. Neoplasia. 2022;32:100822. doi:10.1016/j.neo.2022.100822. | Small prospective pembrolizumab cohort in metastatic castration-resistant prostate cancer with gastrointestinal microbiome profiling. | NOS: Selection 3/4; Comparability 0/2; Outcome 1/3; total 4/9. | Low methodological quality | Very small, highly selected population; concurrent/preceding enzalutamide and heterogeneous prior therapy; limited power, no external validation and substantial multiplicity. | D1 | Hypothesis-generating only. |
| C21 Kim et al., 2024 Kim Y, et al. Fecal microbiota transplantation improves anti-PD-1 inhibitor efficacy in unresectable or metastatic solid cancers refractory to anti-PD-1 inhibitor. Cell Host Microbe. 2024;32:1380–1393.e9. | Prospective single-arm FMT plus continued anti-PD-1 in 13 refractory advanced solid-cancer patients with mechanistic profiling. | ROBINS-I: confounding Serious; selection Serious; intervention classification Low; deviations Moderate; missing data Moderate; outcome measurement Moderate; reporting Moderate. | Serious risk of bias | Small uncontrolled cohort, heterogeneous cancers, donor/recipient selection and continued checkpoint therapy; response attribution to FMT is uncertain despite detailed mechanistic observations. | D1; D2; D5 | Proof-of-concept only. |

**Table S2B. Animal study-level judgments (19 distinct reports)**

Each publication appears once. Human or cellular data embedded in an animal-design paper were not entered as additional studies. Most overall judgments were unclear because one or more allocation, housing or blinding domains were not reported sufficiently.

| **Study and full citation** | **Model and eligibility basis** | **SYRCLE domain profile** | **Overall judgment** | **Principal methodological considerations** | **Domain(s)** | **Weight in synthesis** |
| --- | --- | --- | --- | --- | --- | --- |
| A01 Ito et al., 2015 Ito H, et al. Inhibition of inducible nitric oxide synthase enhances the anti-tumor effects of cancer immunotherapy using a TLR7 agonist in mice. Cancer Immunol Immunother. 2015;64:429–436. | Mouse tumour-immunotherapy model combining iNOS inhibition with a TLR7 agonist. | SYRCLE: SG U; BC L; AC U; RH U; BP U; RO U; BO U; ID L; SR L; OB L. | Unclear overall risk | Direct intervention and immune endpoints; small experimental groups and incomplete reporting of randomization, concealment and blinding; indirect to ICI. | D3; D5 | Supportive context-dependent NO evidence. |
| A02 Ridnour et al., 2015 Ridnour LA, et al. NOS inhibition modulates immune polarization and improves radiation-induced tumor growth delay. Cancer Res. 2015;75:2788–2799. | Mouse tumour models assessing NOS inhibition, immune polarization and radiation response. | SYRCLE: SG U; BC L; AC U; RH U; BP U; RO U; BO U; ID L; SR L; OB L. | Unclear overall risk | Mechanistic immune and tumour endpoints with relevant controls; allocation, blinding and complete exclusion reporting were not fully described; indirect to ICI. | D3; D4 | Supportive NO-immunology evidence; indirect to checkpoint blockade. |
| A03 Sivan et al., 2015 Sivan A, et al. Commensal Bifidobacterium promotes antitumor immunity and facilitates anti-PD-L1 efficacy. Science. 2015;350:1084–1089. doi:10.1126/science.aac4255. | Syngeneic melanoma models comparing microbiota states, fecal transfer and Bifidobacterium administration with anti-PD-L1. | SYRCLE: SG U; BC L; AC U; RH U; BP U; RO U; BO U; ID L; SR L; OB L. | Unclear overall risk | Multiple controls and mechanistic immune assays; randomization, concealment and blinded tumour measurement were not fully described. | D1; D2; D5 | Strong causal preclinical support. |
| A04 Vétizou et al., 2015 Vétizou M, et al. Anticancer immunotherapy by CTLA-4 blockade relies on the gut microbiota. Science. 2015;350:1079–1084. doi:10.1126/science.aad1329. | Multiple syngeneic tumour models, germ-free/antibiotic perturbation and defined bacterial reconstitution during CTLA-4 blockade. | SYRCLE: SG U; BC L; AC U; RH U; BP U; RO U; BO U; ID L; SR L; OB L. | Unclear overall risk | Robust biological controls and replication across models; sequence generation, allocation concealment, random housing and blinded outcome assessment were incompletely reported. | D1; D2; D5 | Strong preclinical support, tempered by reporting limitations. |
| A05 Fauskanger et al., 2018 Fauskanger M, Haabeth OAW, Skjeldal FM, Bogen B, Tveita AA. Tumor killing by CD4+ T cells is mediated via induction of inducible nitric oxide synthase-dependent macrophage cytotoxicity. Front Immunol. 2018;9:1684. doi:10.3389/fimmu.2018.01684. | Syngeneic lymphoma and melanoma models examining antigen-specific CD4 T-cell recognition, macrophage iNOS induction and tumour rejection. | SYRCLE: SG U; BC L; AC U; RH U; BP U; RO U; BO U; ID L; SR L; OB L. | Unclear overall risk | Multiple tumour systems, genetic/pharmacologic perturbation and mechanistic rescue strengthen inference; sequence generation, allocation concealment, random housing and blinded outcome assessment were incompletely reported. | D2; D3; D4 | Strong mechanistic animal evidence for macrophage-derived NO in antitumour immunity. |
| A06 Sung et al., 2019 Sung YC, et al. Delivery of nitric oxide with a nanocarrier promotes tumour vessel normalization and potentiates anti-cancer therapies. Nat Nanotechnol. 2019;14:1160–1169. | Multiple tumour models with localized NO nanocarrier delivery, perfusion/vascular measurements and combination therapy. | SYRCLE: SG L; BC L; AC U; RH L; BP U; RO L; BO U; ID L; SR L; OB L. | Unclear overall risk | Controlled formulation, multiple models, imaging and orthogonal vascular/therapeutic endpoints; some blinding details were incomplete, but the design and replication were comparatively strong. | D3; D4; D5 | High-weight preclinical vascular evidence; not microbiota-derived NO. |
| A07 Tanoue et al., 2019 Tanoue T, et al. A defined commensal consortium elicits CD8 T cells and anti-cancer immunity. Nature. 2019;565:600–605. doi:10.1038/s41586-019-0878-z. | Gnotobiotic mice colonized with a defined 11-strain consortium; immune phenotyping, infection and tumour challenge models. | SYRCLE: SG L; BC L; AC U; RH L; BP U; RO L; BO U; ID L; SR L; OB L. | Unclear overall risk | Defined exposure, appropriate germ-free controls, replicated functional assays and orthogonal validation. Several procedural domains, particularly allocation concealment and blinding, were not reported sufficiently to support a low-risk judgment. | D2; D5 | High-weight preclinical mechanistic evidence. |
| A08 Coutzac et al., 2020 Coutzac C, et al. Systemic short-chain fatty acids limit antitumor effect of CTLA-4 blockade in hosts with cancer. Nat Commun. 2020;11:2168. doi:10.1038/s41467-020-16079-x. | Mouse tumour models testing systemic SCFAs during CTLA-4 blockade, integrated with patient biomarker observations. | SYRCLE: SG U; BC L; AC U; RH U; BP U; RO U; BO U; ID L; SR L; OB L. | Unclear overall risk | Dose-controlled interventions and mechanistic immune analyses; incomplete reporting of allocation, blinding and all exclusions. Results caution against assuming uniformly beneficial SCFA effects. | D2; D5 | Important contradictory/context-dependent preclinical evidence. |
| A09 Shi et al., 2020 Shi L, et al. Combining IL-2-based immunotherapy with commensal probiotics produces enhanced antitumor immune response and tumor clearance. J Immunother Cancer. 2020;8:e000973. | Syngeneic tumour models combining commensal probiotics with IL-2-based immunotherapy. | SYRCLE: SG U; BC L; AC U; RH U; BP U; RO U; BO U; ID L; SR L; OB L. | Unclear overall risk | Appropriate treatment comparators and immune endpoints; randomization, allocation concealment, blinded tumour measurement and attrition reporting were incomplete. | D2; D5 | Supportive intervention evidence; indirect to ICI. |
| A10 Xu et al., 2020 Xu J, Luo Y, Yuan C, Han L, Wu Q, Xu L, et al. Downregulation of nitric oxide collaborated with radiotherapy to promote anti-tumor immune response via inducing CD8+ T-cell infiltration. Int J Biol Sci. 2020;16:1563–1574. doi:10.7150/ijbs.41653. | Murine tumour models combining NO pathway suppression with radiotherapy and assessment of CD8 T-cell infiltration and tumour control. | SYRCLE: SG U; BC L; AC U; RH U; BP U; RO U; BO U; ID L; SR L; OB L. | Unclear overall risk | Relevant intervention controls and immune endpoints were reported; incomplete description of allocation concealment, random housing, blinding and exclusion handling limits certainty. | D3; D4; D5 | Supportive context-dependent NO and immune-access evidence; indirect to ICI. |
| A11 Fan et al., 2021 Fan L, et al. A. muciniphila suppresses colorectal tumorigenesis by inducing TLR2/NLRP3-mediated M1-like tumour-associated macrophages. Cancer Immunol Res. 2021;9:1111–1124. | Colorectal tumour models with A. muciniphila administration, macrophage phenotyping and pathway perturbation. | SYRCLE: SG U; BC L; AC U; RH U; BP U; RO U; BO U; ID L; SR L; OB L. | Unclear overall risk | Mechanistic genetic/pharmacologic controls strengthen inference; randomization, cage effects and blinded histopathology/tumour assessment incompletely reported. | D1; D2; D3 | Supportive mechanistic animal evidence. |
| A12 Luu et al., 2021 Luu M, et al. Microbial short-chain fatty acids modulate CD8+ T cell responses and improve adoptive immunotherapy for cancer. Nat Commun. 2021;12:4077. | Mouse tumour/adoptive immunotherapy models with controlled SCFA exposure and CD8 T-cell analyses. | SYRCLE: SG U; BC L; AC U; RH U; BP U; RO U; BO U; ID L; SR L; OB L. | Unclear overall risk | Multiple models, dose-controlled exposures and functional validation; incomplete reporting of allocation concealment and blinding. | D2; D5 | Strong supportive metabolite–T-cell evidence; indirect to ICI. |
| A13 Hezaveh et al., 2022 Hezaveh K, et al. Tryptophan-derived microbial metabolites activate the aryl hydrocarbon receptor in tumour-associated macrophages to suppress anti-tumour immunity. Immunity. 2022;55:324–340.e8. | Multiple tumour models with microbial-metabolite manipulation, AhR perturbation and macrophage-specific validation. | SYRCLE: SG U; BC L; AC U; RH U; BP U; RO U; BO U; ID L; SR L; OB L. | Unclear overall risk | Strong genetic and orthogonal mechanistic validation; randomization/blinding and cage-level allocation were not consistently detailed. | D2 | High-value mechanistic evidence, with standard animal-reporting limitations. |
| A14 Confino et al., 2023 Confino H, et al. Intratumoral administration of high-concentration nitric oxide and anti-mPD-1 treatment improves tumor regression rates and survival in CT26 tumour-bearing mice. Cells. 2023;12:2439. doi:10.3390/cells12202439. | BALB/c bilateral CT26 model testing intratumoral gaseous NO (50,000 ppm) plus systemic anti-mPD-1. | SYRCLE: SG U; BC L; AC U; RH U; BP U; RO U; BO U; ID L; SR H; OB H. | High overall risk | Small treatment groups, randomization/blinding not reported, multiple time-point comparisons and emphasis on non-significant long-term endpoints; highly localized ultra-high-dose NO limits generalizability. | D3; D5 | Preliminary proof-of-concept only. |
| A15 Cunha et al., 2023 Cunha PP, Bargiela D, Minogue E, Krause LCM, Barbieri L, Brombach C, et al. Infiltration of tumors is regulated by T cell-intrinsic nitric oxide synthesis. Cancer Immunol Res. 2023;11:351–363. doi:10.1158/2326-6066.CIR-22-0387. | T-cell-specific Nos2 deletion models, adoptive transfer and tumour-infiltration experiments examining endogenous T-cell NO in antitumour immunity. | SYRCLE: SG U; BC L; AC U; RH U; BP U; RO U; BO U; ID L; SR L; OB L. | Unclear overall risk | Cell-specific genetics, functional rescue and human/murine corroboration are strengths; incomplete reporting of sequence generation, concealment, housing and blinded tumour assessment prevents a low-risk judgment. | D2; D3; D4 | High-value mechanistic animal evidence, interpreted with standard reporting limitations. |
| A16 Zhu et al., 2023 Zhu X, et al. Microbial metabolite butyrate promotes anti-PD-1 antitumor efficacy by modulating T-cell receptor signaling of cytotoxic CD8 T cells. Gut Microbes. 2023;15:2249143. | Mouse tumour models combining butyrate and anti-PD-1, with CD8 T-cell functional and epigenetic analyses. | SYRCLE: SG U; BC L; AC U; RH U; BP U; RO U; BO U; ID L; SR L; OB L. | Unclear overall risk | Relevant combination comparators and pathway assays; incomplete reporting of randomization, blinding and exclusion handling; translational dose equivalence uncertain. | D2; D5 | Direct supportive preclinical ICI evidence. |
| A17 Fang et al., 2024 Fang J, et al. Akkermansia muciniphila improves gastric cancer treatment by modulating the immune microenvironment. Future Microbiol. 2024;19:481–494. | Gastric cancer model testing A. muciniphila and immune-microenvironment changes. | SYRCLE: SG U; BC U; AC U; RH U; BP U; RO U; BO U; ID U; SR H; OB H. | High overall risk | Limited publicly reported allocation/blinding and replication detail; intervention and outcome selection appear exploratory; checkpoint-specific treatment context and selective reporting require caution. | D1; D2 | Hypothesis-generating preclinical evidence. |
| A18 Lan et al., 2024 Lan X, et al. Akkermansia muciniphila might improve anti-PD-1 therapy against HCC by changing host bile acid metabolism. J Gene Med. 2024;26:e3639. | Hepatocellular carcinoma mouse model testing A. muciniphila, bile-acid metabolism and anti-PD-1 response. | SYRCLE: SG U; BC L; AC U; RH U; BP U; RO U; BO U; ID U; SR U; OB U. | Unclear overall risk | Relevant mechanistic measures and comparators; limited detail on randomization, allocation concealment, blinding, exclusions and reproducibility across models. | D1; D2; D5 | Supportive tumour-specific preclinical evidence. |
| A19 Zhu et al., 2024 Zhu Z, et al. Landscape of tumoral ecosystem for enhanced anti-PD-1 immunotherapy by gut Akkermansia muciniphila. Cell Rep. 2024;43. | Integrated mouse tumour, microbiome and tumour-ecosystem analyses testing A. muciniphila with anti-PD-1. | SYRCLE: SG U; BC L; AC U; RH U; BP U; RO U; BO U; ID L; SR L; OB L. | Unclear overall risk | Comprehensive multi-omic and pathway validation; procedural randomization, allocation concealment and blinded outcome assessment are not fully transparent. | D1; D2; D4; D5 | Strong supportive preclinical evidence. |

**Table S2C. In vitro mechanistic study-level judgments (14 distinct reports)**

Eligibility was broadened to include studies directly examining a mechanistic pathway incorporated into the review framework—including immune-cell function, endothelial regulation, SCFA signalling, nitrate–nitrite–NO biology or tumour-cell responses—even when the experimental system did not directly include an ICI.

| **Study and full citation** | **Cell/tissue model and eligibility basis** | **Eight-domain rubric profile** | **Overall judgment** | **Principal methodological considerations** | **Domain(s)** | **Weight in synthesis** |
| --- | --- | --- | --- | --- | --- | --- |
| V01 Segain et al., 2000 Segain JP, et al. Butyrate inhibits inflammatory responses through NF-κB inhibition: implications for Crohn's disease. Gut. 2000;47:397–403. | Intestinal tissue/cellular inflammatory systems treated with butyrate; NF-κB and cytokine endpoints. | Rubric: C Y; R P; M P; E Y; O P; S Y; P U; V P = 5/8. | Some limitations | Consistent anti-inflammatory pathway evidence, but older non-oncology model, limited exposure-response characterization and no ICI-specific phenotype. | D2 (indirect) | Contextual mechanistic support. |
| V02 Menzel et al., 2004 Menzel T, et al. Butyrate inhibits leukocyte adhesion to endothelial cells via modulation of VCAM-1. Inflamm Bowel Dis. 2004;10:122–128. | Endothelial–leukocyte adhesion assays with butyrate exposure. | Rubric: C P; R U; M U; E P; O U; S P; P U; V P = 3/8. | Important limitations | Limited modern reporting of biological replication, exposure characterization and blinded/automated assessment; non-tumour inflammatory model and narrow mechanistic validation. | D4 (indirect) | Illustrative only. |
| V03 Zapolska-Downar et al., 2004 Zapolska-Downar D, et al. Butyrate inhibits cytokine-induced VCAM-1 and ICAM-1 expression in cultured endothelial cells: the role of NF-κB and PPARα. J Nutr Biochem. 2004;15:220–228. | Cytokine-stimulated endothelial cells treated with butyrate; adhesion molecules and NF-κB/PPARα assays. | Rubric: C Y; R P; M P; E Y; O P; S Y; P U; V P = 5/8. | Some limitations | Clear exposure and pathway readouts; limited replication/reporting by modern standards, non-tumour endothelial model and no direct immune-trafficking or ICI outcome. | D4 (indirect) | Mechanistic plausibility only. |
| V04 Miller et al., 2005 Miller SJ, et al. Short-chain fatty acids modulate gene expression for vascular endothelial cell adhesion molecules. Nutrition. 2005;21:740–748. | Cultured vascular endothelial cells exposed to SCFAs; adhesion-molecule expression endpoints. | Rubric: C Y; R P; M P; E Y; O P; S Y; P U; V P = 5/8. | Some limitations | Appropriate cell controls and quantitative endpoints; older study, non-tumour endothelium, limited orthogonal validation and no ICI context. | D4 (indirect) | Mechanistic plausibility only. |
| V05 Nagaraj et al., 2007 Nagaraj S, Gupta K, Pisarev V, Kinarsky L, Sherman S, Kang L, et al. Altered recognition of antigen is a mechanism of CD8+ T-cell tolerance in cancer. Nat Med. 2007;13:828–835. doi:10.1038/nm1609. | MDSC–T-cell coculture, peptide–MHC binding and biochemical nitration analyses examining peroxynitrite-mediated modification of the TCR–CD8 complex. | Rubric: C Y; R Y; M Y; E Y; O Y; S Y; P Y; V Y = 8/8. | Most criteria met | Appropriate cellular controls, contact-dependence experiments, chemical scavenging and structural/biochemical validation support specificity; translation to ICI-treated human tumours remains indirect. | D2; D3 | High-value mechanistic evidence for reactive nitrogen species-mediated T-cell dysfunction. |
| V06 Molon et al., 2011 Molon B, Ugel S, Del Pozzo F, Soldani C, Zilio S, Avella D, et al. Chemokine nitration prevents intratumoral infiltration of antigen-specific T cells. J Exp Med. 2011;208:1949–1962. doi:10.1084/jem.20101956. | Biochemical chemokine-nitration assays, T-cell chemotaxis systems and human/murine tumour material examining reactive nitrogen species-mediated exclusion of tumour-specific T cells. | Rubric: C Y; R Y; M Y; E Y; O Y; S Y; P Y; V Y = 8/8. | Most criteria met | Defined nitrated chemokine species, functional chemotaxis assays, pharmacologic reversal and orthogonal tumour validation support specificity; ICI response was not directly tested. | D3; D4 | High-value mechanistic evidence linking reactive nitrogen species to immune exclusion. |
| V07 Mundy-Bosse et al., 2011 Mundy-Bosse BL, Lesinski GB, Jaime-Ramirez AC, Benninger K, Khan M, Kuppusamy P, et al. Myeloid-derived suppressor cell inhibition of the IFN response in tumour-bearing mice. Cancer Res. 2011;71:5101–5110. doi:10.1158/0008-5472.CAN-10-2670. | Ex vivo and in vitro MDSC–immune-cell systems assessing NO-dependent impairment of IFN signalling, including Nos2-deficient and pharmacologic controls. | Rubric: C Y; R Y; M Y; E Y; O P; S Y; P Y; V Y = 7/8. | Most criteria met | Genetic and pharmacologic perturbation, quantitative signalling assays and independent validation support mechanism; the model addresses tumour-associated immune suppression rather than direct ICI exposure. | D2; D3 | High-value mechanistic evidence for NO-mediated impairment of interferon responsiveness. |
| V08 Jiang et al., 2012 Jiang H, et al. Concentration- and stage-specific effects of nitrite on colon cancer cell lines. Nitric Oxide. 2012;26:267–273. | Colon cancer cell lines exposed to graded nitrite concentrations across disease-stage phenotypes; proliferation/invasion-related outcomes. | Rubric: C Y; R Y; M P; E Y; O P; S Y; P U; V P = 5/8. | Some limitations | Dose-response design and stage comparison are strengths; cell-line-only system, nitrite-to-NO flux not spatially measured, and no immune or ICI component. | D3 | Supports biphasic/context-dependent nitrite biology only. |
| V09 Raber et al., 2014 Raber PL, Thevenot P, Sierra R, Wyczechowska D, Halle D, Ramirez ME, et al. Subpopulations of myeloid-derived suppressor cells impair T-cell responses through independent nitric oxide-related pathways. Int J Cancer. 2014;134:2853–2864. doi:10.1002/ijc.28622. | Tumour-derived granulocytic and monocytic MDSC cocultures with activated T cells, using NO scavenging, iNOS knockdown and peroxynitrite pathway interrogation. | Rubric: C Y; R Y; M Y; E Y; O P; S Y; P Y; V Y = 7/8. | Most criteria met | Subset-specific comparisons, pathway-selective perturbation and functional T-cell endpoints strengthen causal interpretation; experimental systems remain ex vivo and model-dependent. | D2; D3 | High-value mechanistic evidence for distinct NO-related immunosuppressive pathways. |
| V10 Lu et al., 2015 Lu G, et al. Myeloid cell-derived inducible nitric oxide synthase suppresses M1 macrophage polarization. Nat Commun. 2015;6:6676. | Primary/derived myeloid cells; iNOS manipulation, IRF5 nitration and macrophage-polarization assays. | Rubric: C Y; R Y; M Y; E Y; O Y; S Y; P Y; V Y = 8/8. | Most criteria met | Appropriate genetic/pharmacologic perturbation, controls and orthogonal validation; some exposure-to-clinical-context extrapolation remains indirect. | D2; D3 | High-value mechanistic evidence. |
| V11 Bolduc et al., 2017 Bolduc JF, et al. Epigenetic metabolite acetate inhibits class I/II histone deacetylases, promotes histone acetylation, and increases HIV-1 integration in CD4+ T cells. J Virol. 2017;91:e00524-17. | Cultured CD4 T cells exposed to acetate; HDAC inhibition, histone acetylation and transcriptional outcomes. | Rubric: C Y; R Y; M P; E Y; O P; S Y; P U; V P = 5/8. | Some limitations | Controlled exposure and molecular endpoints, but non-oncology/non-ICI model and limited direct validation of tumour-relevant outcomes. | D2 (indirect) | Contextual mechanistic support only. |
| V12 Li et al., 2018 Li M, et al. The anti-inflammatory effects of short-chain fatty acids on LPS- or TNF-α-stimulated endothelial cells via activation of GPR41/43 and inhibition of HDACs. Front Pharmacol. 2018;9:533. | Stimulated endothelial-cell systems exposed to SCFAs with receptor and HDAC pathway analyses. | Rubric: C Y; R Y; M P; E Y; O P; S Y; P U; V Y = 6/8. | Some limitations | Defined exposures, multiple controls and mechanistic endpoints; non-tumour model and incomplete direct linkage to NO, vascular normalization or ICI response. | D2; D4 (indirect) | Supportive mechanistic evidence. |
| V13 Stiff et al., 2018 Stiff A, Trikha P, Mundy-Bosse B, McMichael E, Mace TA, Benner B, et al. Nitric oxide production by myeloid-derived suppressor cells plays a role in impairing Fc receptor-mediated natural killer cell function. Clin Cancer Res. 2018;24:1891–1904. doi:10.1158/1078-0432.CCR-17-0691. | Human and murine MDSC–NK-cell cocultures assessing NO-dependent impairment of Fc receptor signalling and antibody-dependent cellular cytotoxicity. | Rubric: C Y; R Y; M Y; E Y; O P; S Y; P Y; V Y = 7/8. | Most criteria met | Cross-species validation, NOS inhibition, functional cytotoxicity assays and clinical specimens support the mechanism; relevance is to antibody-mediated immunity rather than direct checkpoint blockade. | D2; D3 | High-value mechanistic evidence for NO-mediated suppression of innate antitumour effector function. |
| V14 Wang et al., 2020 Wang Y, et al. Butyrate mitigates TNF-α-induced attachment of monocytes to endothelial cells. J Bioenerg Biomembr. 2020;52:247–256. | TNF-α-stimulated endothelial cells and monocyte-adhesion assays with butyrate. | Rubric: C Y; R Y; M P; E Y; O P; S Y; P U; V Y = 6/8. | Some limitations | Defined stimulation, functional adhesion endpoint and quantitative analyses; non-tumour model and limited demonstration that changes improve tumour immune-cell trafficking. | D4 (indirect) | Supportive mechanistic evidence. |

**Interpretive notes**

1. The table contains 54 distinct primary reports. No publication is counted in more than one evidence stratum.

2. Overall judgments address the risk that design, conduct or reporting could materially distort the result used in this review; they do not reflect journal prestige or biological importance.

3. Moderate-quality clinical associations may inform consistency and hypothesis generation but should not independently support causality or therapeutic efficacy.

4. Single-arm microbiota-intervention studies were retained as proof-of-concept and judged at serious risk of bias because they lacked concurrent controls and were vulnerable to selection and confounding.

5. An unclear SYRCLE judgment most often reflects incomplete reporting rather than demonstrated invalidity. It should nevertheless reduce evidentiary weight.

6. In vitro studies were used to establish pathway plausibility and specificity, not clinical efficacy. Pharmacologic or ultra-high-concentration NO interventions were not treated as equivalent to physiologic or microbiota-supported nitrate–nitrite–NO production.

The reconstructed inventory and appraisal were cross-checked against Supplementary Table S1, the revised reference list and the PRISMA inventory. Because the original reviewer-specific forms were unavailable, no historical inter-rater agreement statistic is reported.
